# Patient‐derived renal cell carcinoma organoids for personalized cancer therapy

**DOI:** 10.1002/ctm2.970

**Published:** 2022-07-08

**Authors:** Zhichao Li, Haibo Xu, Lei Yu, Jia Wang, Qian Meng, Hongbing Mei, Zhiming Cai, Wei Chen, Weiren Huang

**Affiliations:** ^1^ Department of Urology Shenzhen Institute of Translational Medicine Shenzhen Second People's Hospital The First Affiliated Hospital of Shenzhen University International Cancer Center Shenzhen University School of Medicine Shenzhen China; ^2^ Shenzhen Institute of Synthetic Biology Shenzhen Institutes of Advanced Technology Chinese Academy of Sciences Shenzhen China; ^3^ Key Laboratory of Medical Reprogramming Technology Shenzhen Second People's Hospital The First Affiliated Hospital of Shenzhen University Shenzhen China

**Keywords:** drug screening, organoids, personalized medicine, renal cell carcinoma

## Abstract

**Background:**

Kidney cancer is one of the most common solid tumors. The advancement of human kidney cancer research and treatment has been hindered by a lack of research models that faithfully recapitulate the diversity of the disease.

**Methods:**

We established an effective three‐dimensional culture system for generating kidney cancer organoids from clinical renal cell carcinoma samples. Renal cell carcinoma (RCC) organoids were characterized by H&E staining, immunofluorescence, whole‐exome sequencing, RNA sequencing and single‐cell RNA sequencing. The use of RCC organoids in personalized cancer therapy was assessed by testing their responses to treatment drugs and chimeric antigen receptor T cells.

**Results:**

Using this organoid culture system, 33 kidney cancer organoid lines from common kidney cancer subtypes, including clear cell renal cell carcinoma (ccRCC), papillary renal cell carcinoma (pRCC), and chromophobe renal cell carcinoma (chRCC), were generated. RCC organoids preserved the histological architectures, mutational landscapes, and transcriptional profile of the parental tumor tissues. Single‐cell RNA‐sequencing revealed inter‐ and intra‐tumoral heterogeneity in RCC organoids. RCC organoids allowed for in vitro drug screening and provided a tool for assessing the efficacy of chimeric antigen receptor T cells.

**Conclusions:**

Patient‐derived RCC organoids are valuable pre‐clinical models for academic research and personalized medicine.

## Graphical abstract

- A biobank of patient‐derived renal cell carcinoma organoids was established using a modified culture system.
- RCC organoids maintain cellular heterogeneity, histological, genomic and transcriptomic characteristics of parental tumours.
- RCC organoids allow drug testing and can be used in assessing responses to CAR‐T therapies.

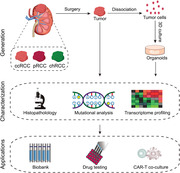

## 1 INTRODUCTION

Kidney cancers, or renal cell carcinomas (RCCs), are a group of histologically defined cancers that can be distinguished by different genetic mutations. Kidney cancer affects an estimated 372 000 people worldwide annually and is responsible for around 166 000 deaths in 2019.^1^ The three major subtypes of RCC are clear cell RCC (ccRCC), papillary RCC (pRCC) and chromophobe RCC (chRCC), which represent 75%, 15% and 5% of RCCs, respectively.^2^, ^3^, ^4^, ^5^

In the clinic, treatment decisions for RCC are usually guided by the disease stages and other factors. Although kidney cancer cells usually do not respond well to chemotherapy drugs, patients with RCC have benefited from several chemotherapy drugs, such as 5‐fluorouracil (5‐FU), gemcitabine and vinblastine.^6^, ^7^, ^8^, ^9^ Overall, chemotherapy is not a standard treatment option for most RCCs due to the unsatisfactory clinical outcomes. One of the main reasons is that it is still impossible to distinguish the patients who warrant chemotherapy from those who will benefit from it. The past decade has seen the approval of several targeted therapeutic drugs for the treatment of RCC. However, the situation in the clinic has not improved much with these targeted agents.^10^, ^11^, ^12^ Among individuals, the outcomes of targeted therapy vary dramatically due to extensive intertumoural heterogeneity.

Precision medicine, or personalized medicine, refers to a medical approach in which each patient is treated based on individual characteristics.^13^ However, the development of personalized medicine for RCC has been hindered by a lack of reliable preclinical models in which the response of candidate treatment regimens can be assessed. Although RCC cell lines, such as the ACHN, A‐498 and Caki‐1 cell line, have improved our knowledge of kidney cancer pathophysiology, they fail to preserve the patients’ genetic backgrounds and tumours’ three‐dimensional (3D) structure. Patient‐derived xenograft (PDX) models preserve tumours’ genetic characteristics and structures. However, PDX models are technically challenging, labour intensive, and costly. Also, the extensive presence of murine viral infection in PDXs altered the expression of many genes and may affect tumour cells’ response to treatments.^14^ Therefore, a suitable research model of RCC that faithfully represents this disease and allows for drug testing is eagerly pursued.

The past decade has witnessed the rapid development of organoid technology. Organoids are 3D, stem‐cell‐derived, self‐organized miniature tissues that recapitulate the structure and functionality of their parental tissue counterparts.^15^, ^16^, ^17^ The establishments of tumour organoids have been reported for the most common types of cancers, including prostate cancer, colorectal cancer, pancreatic cancer, liver cancer, breast cancer, bladder cancer, gastric cancer, ovarian cancer and endometrial cancer.^18^, ^19^, ^20^, ^21^, ^22^, ^23^, ^24^, ^25^, ^26^ Two recent studies reported the establishment of RCC organoid lines from clinical samples, but these organoid lines have not been extensively characterized.^27^, ^28^ Another study recently developed a protocol for the culture of childhood kidney cancer organoids and established the first paediatric cancer organoid biobank.^29^ Paediatric kidney cancer organoids described in this study were extensively characterized.^29^ However, substantial genetic and pathological differences existed between childhood and adult kidney cancers.^30^

Here, we established a culture system to generate tumour organoids using adult RCC tissues. Using this system, we successfully derived 33 RCC organoid lines and 10 normal kidney organoid lines. Next, we provide a thorough characterization of RCC organoids, including histopathological characteristics, mutational landscape, global gene expression profile and cellular heterogeneity. Finally, RCC organoid lines were used to assess the responses of engineered chimeric antigen receptor (CAR)‐T cells and treatment drugs.

## 2 RESULTS

### 2.1 Establishment of patient‐derived RCC organoids

**FIGURE 1:**
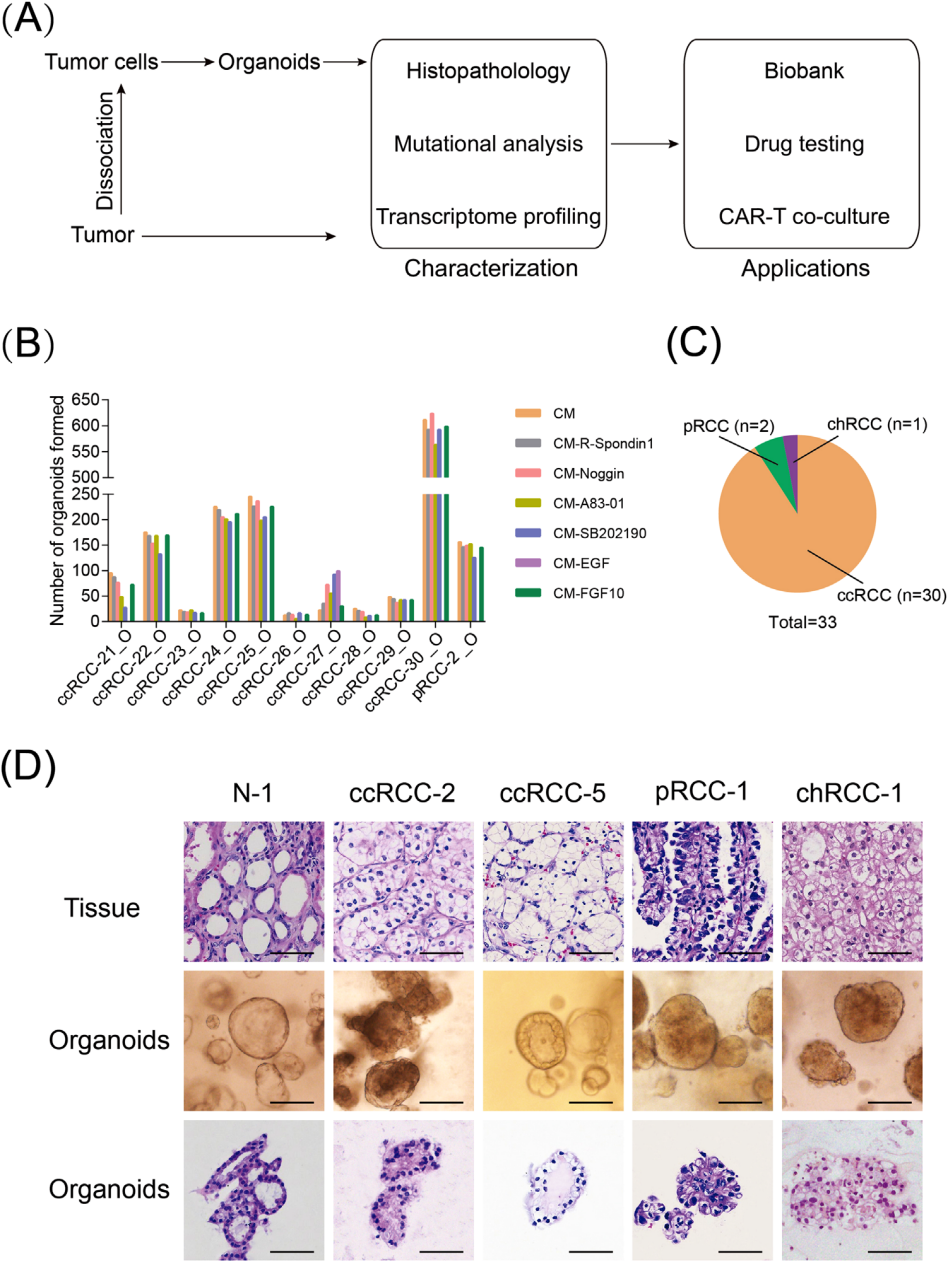
Establishing a biobank of patient‐derived renal cell carcinoma (RCC) organoids: (A) overview of experimental design; (B) RCC organoid formation efficiency in basal medium (BM) and modified medium (with each component individually omitted from the BM), shown are bright‐field images of RCC organoids formed after 2 weeks of culture in indicated media. Scale bar, 100 μm; (C) pie chart showing the subtypes of established 33 RCC organoids in this study; (D) representative haematoxylin–eosin (H&E) staining images of RCC tumour tissue (top row) together with the bright‐field microscopy images (middle row) and H&E staining images (bottom row) of corresponding RCC organoids. Scale bar, 50 μm

Resected RCC tissues were digested, and tumour cells were mixed with cold basement membrane extract (Matrigel) and plated for organoid culture (Figure 1A). Patient information is summarized in Table S1. Classical culture media for cancer organoids usually contains the following niche factors: B‐27 supplement, nicotinamide, R‐spondin1, noggin, *N*‐acetyl‐l‐cysteine, A83‐01, SB202190; fibroblast growth factor (FGF) 10, epidermal growth factor (EGF) and Y‐27632.^21^, ^22^, ^24^, ^31^

To improve the success rate of RCC organoid derivation, we tested the effect of these niche factors on the formation of RCC organoids. Each niche factor was individually omitted from the culture medium, as shown in Figure 1B. We noted that the number of tumour organoids formed significantly varied among different samples, and RCC organoid lines showed similar requirements for the following medium components: (1) EGF was required for the formation of all the RCC organoid lines except ccRCC‐27_O (Figure S1). This was in line with the results that the EGF receptor and the corresponding signalling pathways are highly enriched in kidney cancer cells (Figure S2A–C); (2) A83‐01, R‐spondin1, noggin and FGF10 were not required for all tested RCC organoids and, therefore, were excluded from the medium; (3) the omission of SB202190 caused RCC organoids to display hollow and cystic structures in ccRCC‐21_O and ccRCC‐29_O (Figure S1). To avoid the potential overgrowth by nontumoural organoids, we also tested the niche factor requirements by normal kidney organoids using a similar strategy. For the six tested samples, we found that the depletion of R‐spondin1 reduced the growth of normal kidney organoids (Figure S3A,B). This observation was in agreement with our findings that WNT‐ and stemness‐associated signalling pathways were highly activated in normal kidney organoids (Figure S4A–C) and may sustain tissue self‐renewal. It was also observed that the depletion of A83‐01 reduced the growth of normal kidney organoids (Figure S3A,B). To be noted, normal kidney organoids were generated from tumour‐adjacent tissues. Although these tissues displayed histological characteristics of normal tissues, the unique gene expression profiles differentiate them from truly normal tissues.^32^ In summary, RCC organoids and normal kidney organoids demonstrate different requirements for niche factors, and the culture medium composition for RCC organoids and normal kidney organoids is summarized in Table S2.

RCC organoids were passaged every 2–3 weeks with a split ratio of 1:2–1:3. Using this organoid culture system, we successfully generated 33 RCC organoid lines from 43 donors with common types of RCC (Figure 1C). Seventy per cent (30/43) of the patients were male, consistent with the predominant incidence of RCC in men over women.^33^ We also derived 10 normal kidney organoid lines from tumour‐adjacent tissues.

### 2.2 RCC organoids maintain the histopathological characteristics of original tumours

We performed haematoxylin–eosin (H&E) staining to test whether RCC organoids maintain the histopathological characteristics of their parental tumours. The results revealed that RCC organoids preserved the histological patterns of their parental tumours. For example, ccRCC‐5_O displayed classic ccRCC features, such as clear cytoplasm and distinct but delicate cell boundaries (Figure 1D). The chRCC‐1_O line showed large pale cells with perinuclear halos and reticulated cytoplasm, consistent with its parental tumour tissue (Figure 1D). In contrast, normal kidney organoids presented a well‐organized, glandular and single‐cell‐layered structure (Figure 1D).

**FIGURE 2:**
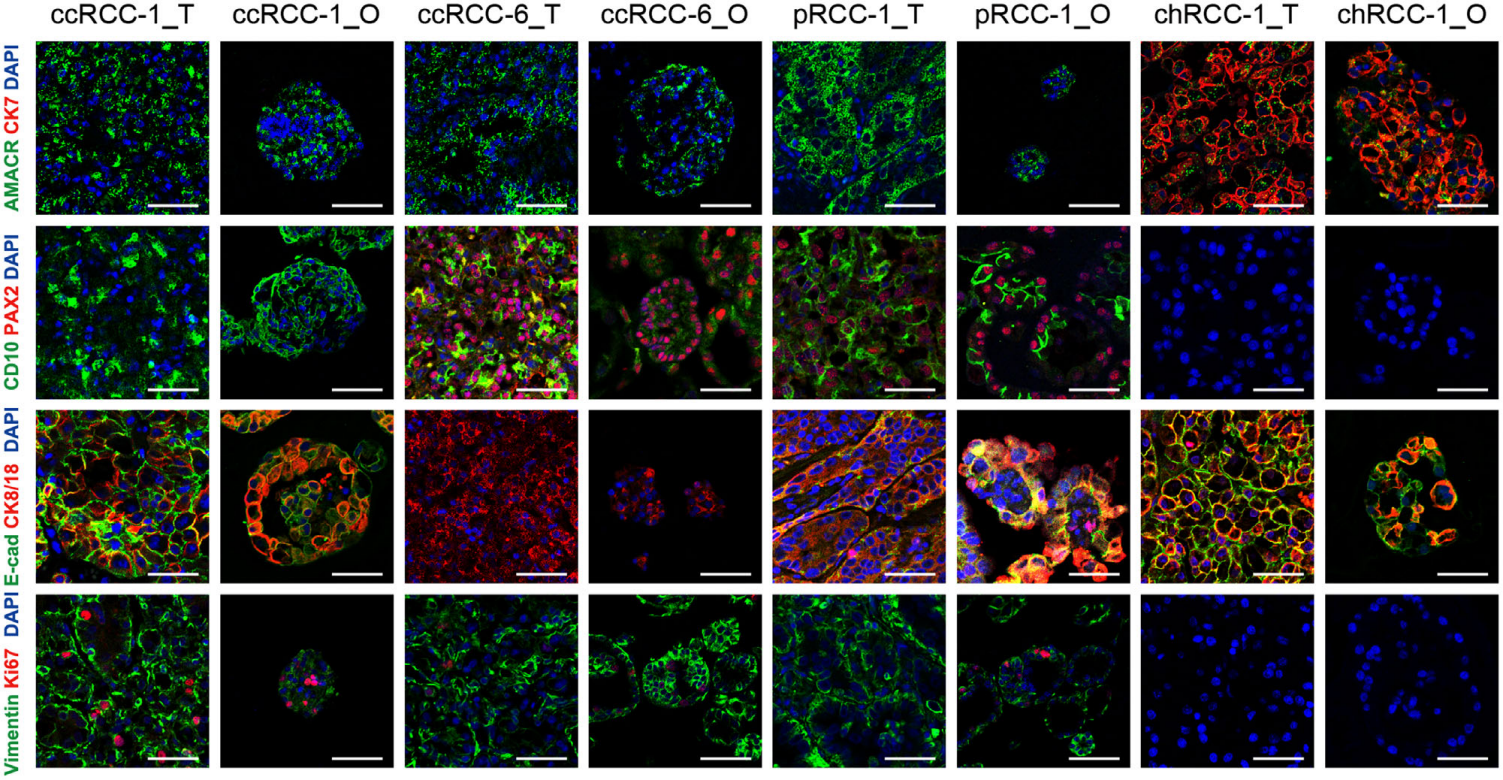
Patient‐derived renal cell carcinoma (RCC) organoids preserve histopathological characteristics of parental tumours. Representative immunofluorescence staining images of paired RCC tumours and organoids (_T, tumour; _O, organoids) for alpha‐methylacyl CoA racemase (AMACR), Cytokeratin 7 (CK7), CD10, PAX2, E‐cadherin, CK8/18, vimentin and Ki‐67. Nuclei were stained with 4′,6‐diamidino‐2‐phenylindole (DAPI) (blue). Scale bar, 50 μm

Next, we examined the expression pattern of RCC subtype‐specific markers in RCC organoids and matched tumours to further characterize the established RCC organoid lines. Cytokeratin 7 was highly expressed in chRCC‐1_O and its parental tumour but was not detected in tumours and organoids of ccRCC‐1 and pRCC‐1 (Figure 2). CD10 and vimentin were positively stained in ccRCC‐1, ccRCC‐6 and pRCC‐1 tumour–organoid pairs, in agreement with their proximal tubule origin. All the organoids and tumours showed positive staining of CK8/18, consistent with a previous report.^34^ Alpha‐methyl CoA racemase (AMACR) was also expressed in all the RCC tissue‐organoid pairs, and the expression pattern was consistent between tissues and corresponding organoids. We also stained PAX2, E‐cadherin and Ki‐67 in our RCC organoids and parental tumours. The results revealed that each RCC organoid line showed similar expression patterns of these markers with their parental tumours (Figure 2).

### 2.3 RCC organoids preserve the mutational landscape of the corresponding tumours

**FIGURE 3:**
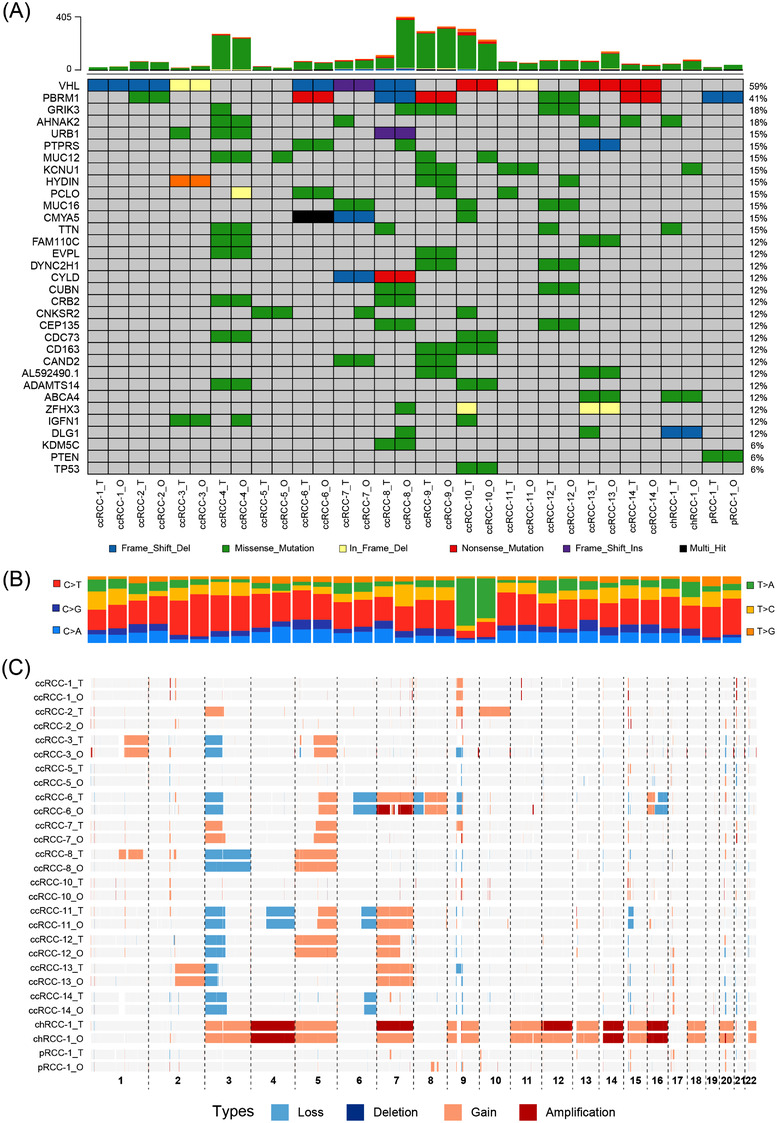
Renal cell carcinoma (RCC) organoids recapitulate the genetic alterations in the parental tumours: (A) the somatic genomic landscape of 16 RCC organoid lines (_O) and the corresponding parental tumours (_T). The types of genetic alterations are indicated in the legend: (B) proportions of base substitutions in RCC organoids (_O) and parental tumours (_T); the six types of base substitutions are represented: (C) DNA copy number alterations in RCC organoids (_O) and tumour tissues (_T).

To test whether RCC organoids preserve the mutational landscape of their parental tumours, whole‐exome sequencing (WES) was performed on 16 established RCC organoids and their matched tumour samples. The shared known RCC‐associated mutations found in the tumours were largely preserved in the corresponding RCC organoids.^3^, ^4^, ^5^ Somatic mutations in VHL, the most frequently mutated gene in RCCs, were identified in 10 RCC organoids and matched tumours (Figure 3A). We also observed somatic mutations in other RCC‐associated genes, such as PBRM1 and AHNAK2, most of which were conserved between RCC organoids and the corresponding tumour tissues (Figure 3A). However, occasional gains or losses of genetic mutations were observed in RCC organoids, such as GRIK3, MUC12, PLCO and TTN (Figure 3A), which may be explained by the intratumour diversification occurs during the expansion of neoplastic cells. The comparative analysis of the WES data revealed that base substitutions in RCC tissues were well retained in their RCC organoids (Figure 3B). In addition, the most and the least frequent base substitutions in RCC tissues and organoids were C>T/G>A transitions (Ti) and T>G/A>C transversions (Tv), respectively, consistent with what was previously described (Figure S5A,B).^3^ Copy number alteration (CNA) analysis demonstrated that DNA gains and losses were also conserved between RCC organoids and tumour tissues (Figure 3C). The most frequent chromosome‐level event in our RCC organoids was the loss of chromosome 3p, which was also seen in the corresponding tumour tissues. Overall, we demonstrate that RCC organoids preserve the mutational landscape of RCC tumours, including somatic mutations, somatic base substitutions and CNAs.

### 2.4 Transcriptional analysis of RCC organoids

**FIGURE 4:**
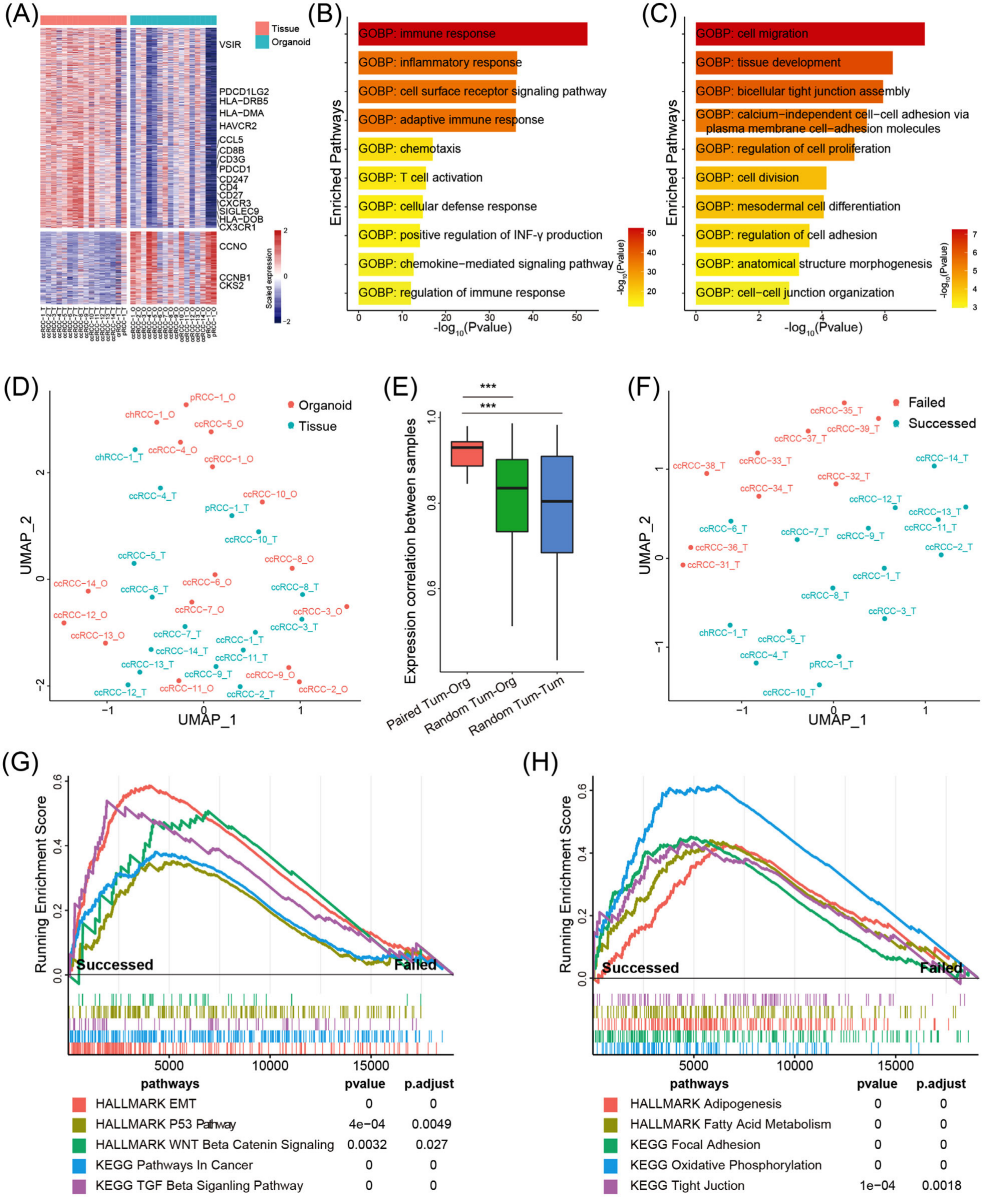
Transcriptomic analysis of renal cell carcinoma (RCC) organoids: (A) Heat map showed the differentially expressed genes between RCC tissues and organoids. Genes with |log_2_FC| > 1 and adjust *p* < .05 were presented. A total of 1353 genes and 491 genes were presented in the upper and lower panel, respectively; (B and C) boxplot showed the top 10 significantly enriched pathways in RCC tissues (B) and organoids (C) using DEGs in DAVID database; (D) Uniform Manifold Approximation and Projection (UMAP) plot of the RNA sequencing (RNA‐seq) data of RCC organoids and tissues; (E) boxplot showed the gene expression correlation between tumour–organoid pairs, random tumour–organoid pairs or random tumour–tumour pairs; (F) UMAP plot of the RNA‐seq data from 16 RCC samples which successfully formed organoids and 10 RCC samples that failed to derive tumour organoids; (G and H) GSEA plot showed the enrichment of cancer‐associated pathways (G) and metabolism/adhesion‐associated pathways (H) between RCC tumours which successfully formed organoids and those failed to derive tumour organoids.

To determine whether our RCC organoids retain the gene expression profile of the original tumours, RNA sequencing (RNA‐seq) was performed on 16 RCC tumour–organoid pairs. Totally, 1844 genes were differentially expressed between tumour tissues and tumour organoids (Figure 4A). Genes that were lowly expressed in RCC organoids, such as CD4, CD8, PDCD1, HLA‐DMA and CX3CR1 were mainly associated with immune response and inflammatory response (Figure 4A,B). This was consistent with the fact that RCC organoids lost the tumour microenvironment (TME) elements (Figure S6A). Highly expressed genes in RCC organoids, including CCNO, CCNB1 and CKS2, were mainly associated with cell division and proliferation (Figure 4A,C). Enrichment analysis based on GSEA method supported that RCC organoids were positively enriched for cell cycle–associated biological processes but negatively enriched for immune‐associated biological processes (Figure S6B,C). Dimension reduction by Uniform Manifold Approximation and Projection (UMAP) showed that RCC tissues and organoids were randomly distributed (Figure 4D), and the correlation analysis of gene expression profiles revealed that each RCC organoid line displayed a higher concordance to its corresponding tumour than those between random tumour–organoid pairs and tumour–tumour pairs (Figure 4E).

To determine the tissue characteristics required for the successful derivation of tumour organoids, we compared transcriptomes of 16 RCC tissues from which tumour organoids have been successfully derived with transcriptomic data of 9 RCC tissues being unable to derive tumour organoids. UMAP analysis revealed distinct transcriptomic differences between the two groups of RCC samples (Figure 4F). Differential expression analysis also revealed dramatic differences between these two groups at transcription levels (Figure S6D). RCC tissues that successfully generated organoids highly expressed genes associated with stemness (WNT6/11, FZD8/9 and JAG2), cell matrix (MMP2, COL1A1), fatty metabolism (ACOT2, LIPE) and cancer development (TGFB1, SMAD6/7, EGF and FGF1) (Figure S6D). GSEA and DAVID enrichment analyses also demonstrated that tumour tissues that could form RCC organoids were highly enriched for pathways associated with EMT, WNT, fatty acid metabolism and focal adhesion (Figures 4G,H and S6E,G). RCC samples which could not form the RCC organoids highly expressed genes associated with DNA repair and recombination (Figure S6F,G), suggesting these 9 RCC samples might suffer more DNA damage. Those data suggest that enrichment of stemness‐related properties and genomic stability might be essential for the successful establishment of RCC organoids.

### 2.5 RCC organoids allow for the identification of potential tumour biomarkers

We compared the RNA‐seq data of RCC organoids to those of normal kidney organoids (N‐1_O, N‐2_O, N‐3_O and N‐4_O) to assess the potential of RCC organoids as a platform for identifying tumour biomarkers. Among the differentially expressed genes between RCC organoids and normal kidney organoids, 30 upregulated genes and 30 downregulated genes with the lowest *p*‐values were used for further analysis. Among these genes, 20 genes were previously shown to be upregulated, and 7 genes were previously reported to be downregulated in RCC, including SOX2, NDUFA4L2, C1QA, C1QB and C1QC (Figure S7A). We then assessed the prognostic values of the remaining genes by performing survival analysis based on cox proportional‐hazards model using TCGA data. In KIRC cohort, both uni‐ and multi‐Cox regression analyses showed that overexpression of ADGRF5, EVC, GCHFR, GIMAP1/6 and HLF conferred good prognosis, whereas the overexpression of GOLGA8A, HS3ST4, LINC00173, MIA, PABPC1L and RRP7BP predicted poor prognosis (Figure S7B,C). Other genes, such as GIMAP5, HIVP3, ROBO4, TPSAB1 and TRMT98 were significantly correlated with prognosis only under uni‐Cox regression analysis (Figure S7B,C). MORC4 and SLC26A2 were significantly correlated with prognosis under both uni‐ and multi‐Cox regression analyses in the KIRP cohort (Figure S7D,E) and KICH cohort (Figure S7F,G), respectively. These results show that RCC organoids could serve as a model to identify potential tumour biomarkers.

### 2.6 scRNA‐seq reveals the cell heterogeneity within RCC organoids

Single‐cell RNA‐seq was performed on three RCC organoid lines to characterize cell heterogeneity. The results revealed that these RCC organoids were mainly composed of epithelial tumour cells (Figure S8A–C). A small portion of TME cells, such as endothelial cells, myofibroblast and immune cells, were also detected (Figure S8A–C). This is not surprising and consistent with a previous report which showed that paediatric kidney cancer organoid cultures contained stromal cells.^29^ It is noteworthy that each RCC organoid line contained distinct components of TME cells and displayed substantial intratumour heterogeneity. For example, endothelial cells were detected in ccRCC‐15_O, and a large cluster of immune cells were preserved in ccRCC‐7_O (Figure S8A–C). Consistent with this, a large number of endothelial cells and fibroblast cells were found in ccRCC‐15 tumour tissue, and ccRCC‐7 tumour revealed high infiltration of CD8‐positive T cells (Figure S9).

**FIGURE 5:**
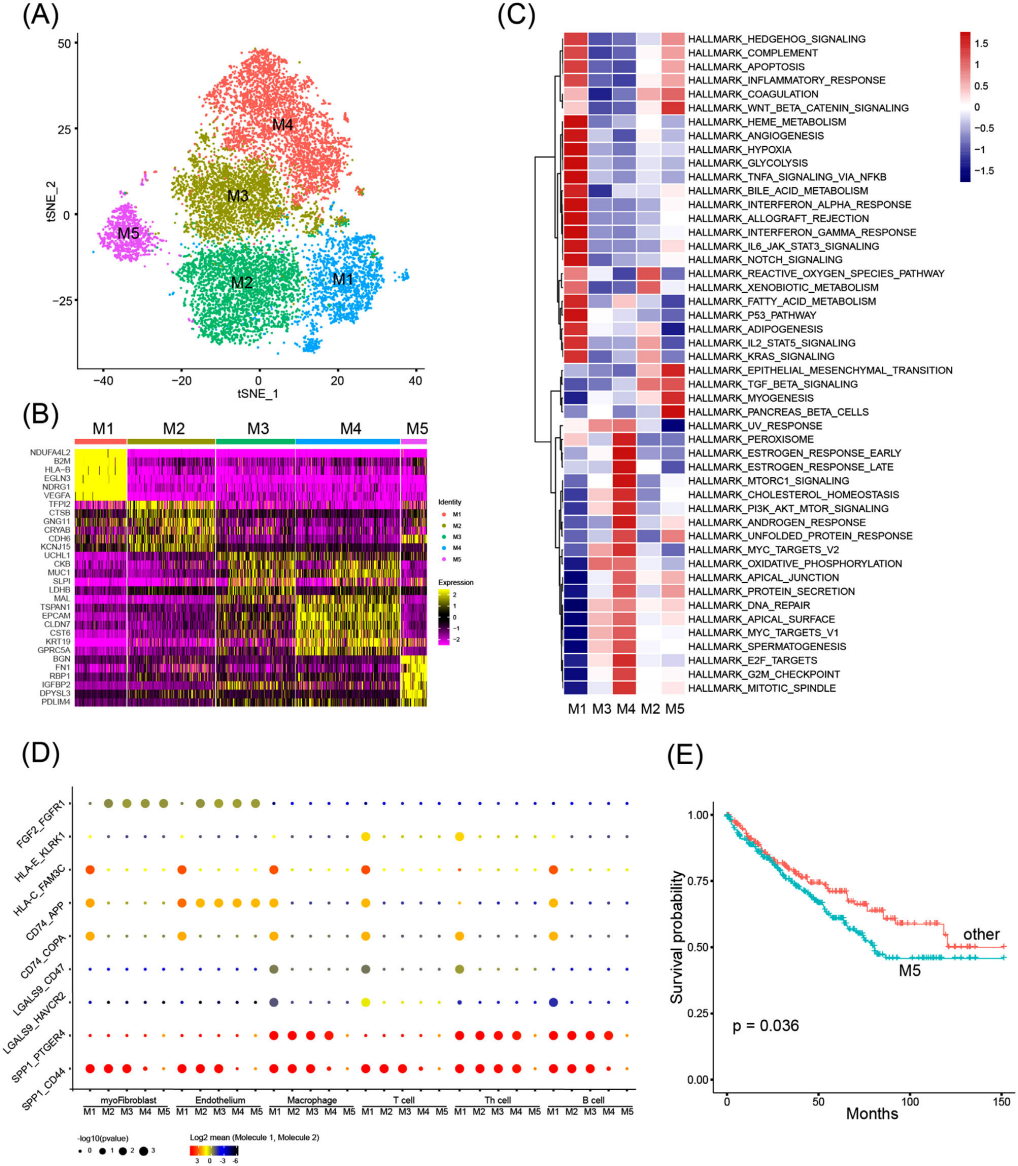
Analysis of cellular heterogeneity in clear cell renal cell carcinoma (ccRCC) organoids by single‐cell RNA sequencing: (A) tSNE plot of 14217 cells from 3 RCC organoid lines. Each dot represents one single cell coloured by cluster identity; (B) heat map showed the expression of marker genes for each subcluster calculated using roc algorithm in FindMarkers module; (C) heat map showed the enrichment of hallmark pathways in each subcluster calculated using VISION; (D) heat map of cell‐type‐specific ligand–receptor interactions inferred by CellPhoneDB. Circle size indicates the significance of interactions and circle colour indicates the mean expression of receptor and ligand genes for each pair; (E) Kaplan–Meier analysis of overall survival (OS) in TCGA cohorts separated by M5 signature using SingleR script

A recent study suggested that tumour cells in RCC biopsies formed two major clusters, tumour programme 1 (TP1) and tumour programme 2 (TP2).^35^ Next, we sought to understand cell programmes active and cell heterogeneity within RCC organoids. The alignment of tumour cells derived from these three RCC organoid lines formed five major clusters, M1–M5 (Figure 5A,B). Cluster M1 highly expressed TP1 markers, and M2–M5 highly expressed TP2 markers (Figure S8D). Gene signatures scored by VISION showed that different gene sets were enriched in each cluster (Figure 5C). Cells in M1 highly expressed gene sets related to angiogenesis, hypoxia, glycolysis, hedgehog, NOTCH and p53 (Figure 5C). In addition, many immune‐associated gene sets were also highly enriched in M1 cells, such as complement, inflammatory and interleukin (Figure 5C). Pathways related to oxidative phosphorylation, mTOR and DNA repair programmes were enriched in M2–M5 cells (Figure 5C). Notably, WNT and EMT processes were highly activated in M2 and M5 cells (Figure 5C). The SCENIC analysis disclosed that cancer cells in each cluster were regulated by different regulons. M2 and M5 subclusters have a similar pattern of regulon activities, and M3 and M4 subclusters share similar regulon activities, all of which were distinct from that of the M1 subcluster (Figure S10A). These data suggest that RCC cells have three different cellular fates.

**FIGURE 6:**
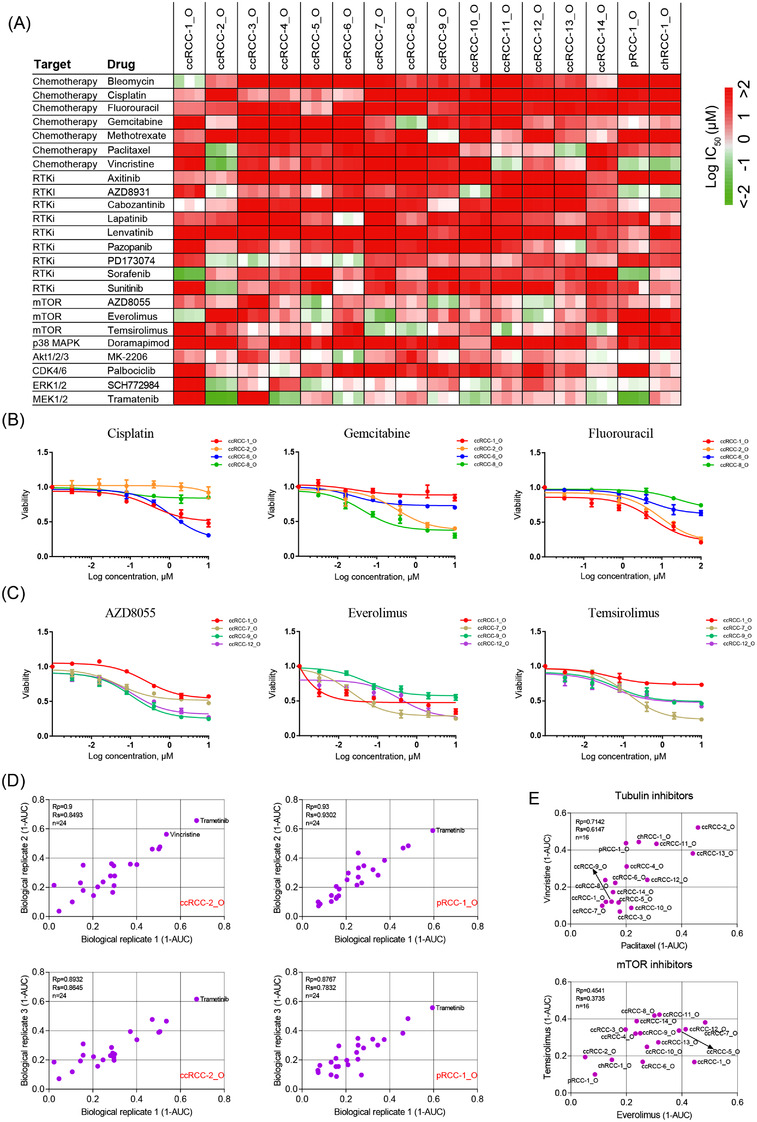
Drug screening in patient‐derived renal cell carcinoma (RCC) organoids: (A) heat map of logIC_50_ values for 24 compounds tested on 16 RCC organoid lines; (B and C) dose–response curves for RCC organoids treated with the indicated chemotherapy drugs (B) and mTOR inhibitors (C). Each data point represents the mean of three biological replicates (organoids from different passages), with error bars representing ± standard error of the mean (SEM); (D) representative scatterplots of 1‐AUC (area under the curve) values for two biological replicates of the drug screening data, highlighting drugs (red) having an obvious inhibitory effect on viability (1‐AUC > .5 for both biological replicates) of indicated organoid lines; (E) representative scatterplots of 1‐AUC from drug screening data of paired drugs with the same nominal targets. Each data point represents three biological replicates, with error bars representing ± SEM

Next, Monocle 2 was used to perform pseudotime analysis to infer the possible cell fate of RCC cells, and the trajectory result revealed three cell branches (Figure S8E). Cells along branch 1 mainly belong to ccRCC‐7_O, whereas cells along branches 2 and 3 mainly existed in ccRCC‐2_O and ccRCC‐15_O (Figure S8C). Drug screening results demonstrated that ccRCC‐2_O and ccRCC‐7_O displayed differential responses to mTOR, ERK and MEK signalling pathway inhibitors (Figure 6A). The analysis of the scRNA‐seq data revealed that target genes in these targeting therapeutics and the corresponding signalling pathways were differentially activated between cancer cell subclusters (Figure S10B,C), which might account for the differences in drug sensitivities to the treatment regimens. Although the mRNA level of mTOR in the M1 subcluster was low (Figure S10B), the mTOR signalling pathway was highly activated (Figure S10C), which may explain the high sensitivity to mTOR inhibitors by ccRCC‐7_O. The MAPK signalling pathway was highly activated in subcluster M2–M5 agreed with the high sensitivity to the specific ERK1/2 inhibitor SCH772984 (Figure S10B,C).

The enrichment of immune‐associated pathways in M1 cells suggests that these cells may exert proper immunoregulatory functions (Figure 5C). To identify the possible interactions between cancer cells and TME cells, CellPhoneDB was utilized to infer putative signalling interactions through known ligand–receptor pairs (Figure 5D). The results showed that M1 cells highly expressed several immune checkpoints (HLA‐C, LGALS9 and SPP1) and evasion (CD74) associated ligands which could interact with the immune cells (Figure 5D). By contrast, the interactions previously mentioned did not exist in cluster M5 (Figure 5D). We also observed a large number of interactions between M2–M5 cells and myofibroblast/endothelium (Figure S8F). Those data revealed different roles of M1 and M2–M5 in influencing the TME.

As multiple signalling pathways and biological processes were differentially enriched in M1–M5, we next investigated whether these clusters predict prognosis. Using SingleR, we utilized our scRNA‐seq results to classify the TCGA cohort into M1‐like, M2‐like and so on. Survival analysis showed an M5‐like cohort, but not others showed significant low survival probability (Figures 5E and S8G–I). These data suggested that RCC cohorts enriched in features of cluster M5 are expected to have a poor prognosis.

### 2.7 Drug responses of RCC organoids

To explore the use of RCC organoids as tumour surrogates to predict responses to treatment regimens, we performed drug screenings on 16 RCC organoid lines. Twenty‐four drugs including chemotherapy drugs and targeted therapy drugs were selected in this study. The responses to drugs of the RCC organoid lines are shown by the half‐maximal inhibitory concentration (IC_50_) and the area under the dose–response curve (AUC).

Consistent with previous reports, RCC organoids were resistant to conventional chemotherapy drugs, as shown by the large IC_50_ values (Figure 6A). Within the organoid lines, the responses to chemotherapy drugs demonstrated striking differences (Figure 6A,B). For example, the ccRCC‐1_O was sensitive to 5‐FU and resistant to gemcitabine, cisplatin and paclitaxel. Gemcitabine was effective for treating ccRCC‐2_O and ccRCC‐8_O and ineffective for ccRCC‐6_O, whereas their responses to cisplatin displayed completely different patterns (Figure 6A,B).

Targeted agents inhibiting the receptor tyrosine kinase (RTK) signalling and mechanistic target of rapamycin complex 1 have been approved for the treatment of RCC. In this study, we assessed the responses of these targeted drugs by established RCC organoids. The drug screening results showed that most of our RCC organoids did not respond well to RTK signalling pathway inhibitors (Figure 6A), which may be due to the lack of the corresponding targets in this in vitro culture system. In contrast, we observed substantial inhibition of organoid formation by mTOR inhibitors everolimus, temsirolimus and AZD8055 (Figure 6A,C). Although most RCC organoids displayed a similar and concordant sensitivity pattern to everolimus, temsirolimus and AZD8055 (Figure 6C), remarkable differences existed in the reactions to these mTOR inhibitors by different RCC organoids. The reason for this difference is unclear, highlighting the value of functional drug tests using patient‐derived RCC organoids. We also found that several targeted agents, which had not been approved for treating RCC, demonstrated promising results for killing RCC organoids. This included AKT inhibitor MK‐2206, MEK1/2 inhibitor trametinib and ERK1/2 inhibitor SCH772984 (Figures 6A and S11).

In addition, a positive correlation was observed between AUC values from biological replicates, suggesting stable and consistent responses to drugs by these RCC organoids (Figures 6D and S12). We also found that the tubulin inhibitors paclitaxel and vincristine, mTOR inhibitors everolimus and temsirolimus demonstrated comparable activity across all RCC organoid lines (Figure 6E).

### 2.8 Assessment of CAR‐mediated cytotoxicity using RCC organoids

Patients with RCC have benefited greatly from immune therapeutics, such as interferon (IFN) and tyrosine kinase inhibitors. CAR‐T‐cell therapy is a novel immune therapy approach, and several clinical trials evaluating the efficacy of CAR‐T therapy for RCC are ongoing (NCT03393936, NCT01218867, NCT02830724 and NCT04438083). To explore the utility of our RCC organoid models in predicting patients’ responses to CAR‐T cells, we adopted a coculture system to incubate RCC organoids with CAR‐T cells.^36^

**FIGURE 7:**
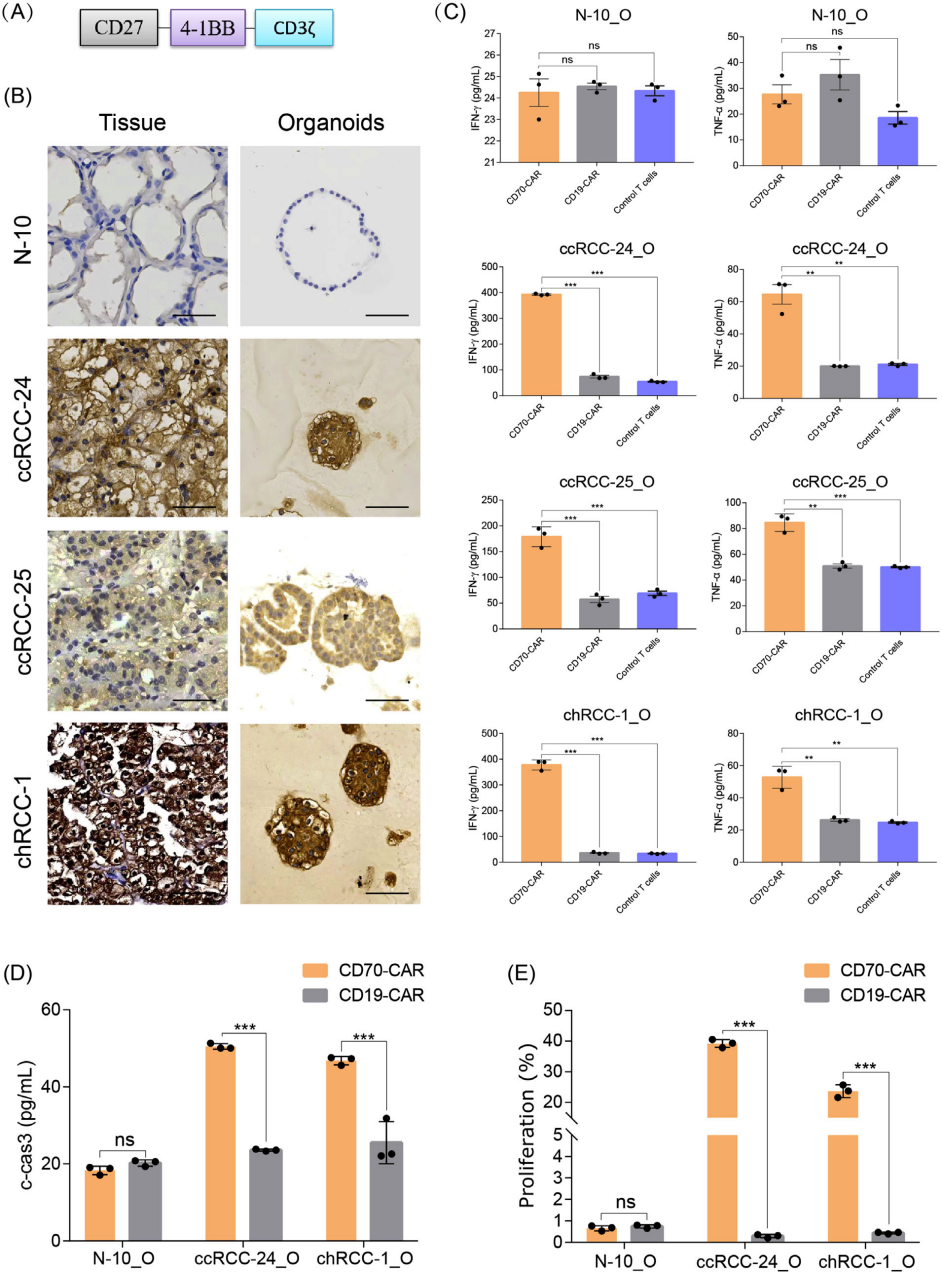
Modelling immunotherapy with coculture of renal cell carcinoma (RCC) organoids and chimeric antigen receptor (CAR)‐T cells: (A) The structure of CD70‐specific CAR; (B) the expression level of CD70 in one normal kidney tissue–organoid pair N‐10, and three RCC tumour–organoid pairs clear cell renal cell carcinoma (ccRCC)‐24, ccRCC‐25 and chromophobe renal cell carcinoma (chRCC)‐1 by immunohistochemistry. Scale bar, 50 μm; (C) quantification of the production of tumour necrosis factor (TNF)‐α and interferon (IFN)‐γ by ELISA at 2 days after coculture of RCC organoids or normal kidney organoids with CD70 CAR‐T cells, CD19 CAR‐T cells or control CAR‐T cells; (D) the level of cleaved‐caspase‐3 in RCC or normal kidney organoids after coculture with CD70 CAR‐T cells, CD19 CAR‐T cells or control CAR‐T cells for 2 days; (E) quantification of the percentage of CFSE (carboxyfluorescein succinimidyl ester)‐labelled T cells after incubation with RCC or normal kidney organoids for 3 days. Values represent mean ± standard error of the mean (SEM) (*n* = 3). ns, not significant. **p < .01; ***p < .001 by two‐tailed, unpaired *t*‐test

The expression profiles of a panel of widely used targets for CAR‐T therapy across 10 RCC tumour samples were obtained via immunohistochemistry. Among these antigens, CD70 was highly expressed in some of the tested samples (Figure S13) and, therefore, was used as the target in this study. The CD70‐specific CAR construct was composed of the full‐length human CD27 (CD70 receptor), the signalling domain of the costimulatory molecule 4‐1BB and the signalling domain of the T‐cell receptor CD3‐zeta chain (Figure 7A).^37^ CD19‐targeting CAR‐T cells and T cells infected with the empty vector were used controls. Three CD70^+^ RCC organoid lines, ccRCC‐24_O, ccRCC‐25_O and chRCC‐1_O, were used to assess the responses of CAR‐T cells. One normal CD70^−^ kidney organoid line N‐10_O which was derived from the adjacent normal kidney tissue of chRCC‐1 was used to assess specificity of CAR‐T cells (Figure 7B).

The production of IFN‐γ and tumour necrosis factor (TNF)‐α was significantly increased when CD70^+^ RCC organoids were incubated with CD70 CAR‐T cells but not control T cells (Figure 7C). In contrast, CD70 CAR‐T cells did not increase the production of IFN‐γ and TNF‐α when cocultured with normal kidney organoids (Figure 7C), demonstrating the high specific activity of CD70 CAR‐T cells. The cleaved Caspase‐3 signal was also increased in CD70^+^ RCC organoids after coculture with CD70 CAR‐T cells (Figure 7D), suggesting that CD70 CAR‐T cells specifically targeted and killed CD70^+^ RCC cells. The coculture with CD70^+^ RCC organoids but not normal kidney organoids significantly increased the proliferation of CD70 CAR‐T cells (Figure 7E). In contrast, CD19 CAR‐T cells did not enter the cell cycle on coculture with tumour cells (Figure 7E). These results demonstrate that the established RCC organoids offer a new platform to assess the efficacy of antigen‐specific CAR‐T cells.

## 3 DISCUSSION

RCCs are cancers originating from the renal epithelium and account for 4% of all cancers.^38^ Currently, the treatment for RCC is guided mainly by the clinical stage.^33^, ^39^ Partial nephrectomy or radical nephrectomy is recommended for patients with localized RCC. For patients with poor performance status or inoperable RCC, systemic therapies should be the suitable option. However, the clinical outcomes of conventional chemotherapy or targeted therapy are often unsatisfactory due to extensive intertumoural heterogeneity.

In this study, we established a library of RCC organoids from resected clinical tissues using a modified culture system. The composition of the RCC organoid culture media was optimized by testing RCC organoids’ dependence on each niche factor. Compared to normal kidney organoids, RCC organoids revealed a loss of niche factor dependence, in agreement with the observations in human pancreatic tumour organoids, colorectal cancer organoids, and lung cancer organoids.^40^, ^41^, ^42^ The removal of R‐spondin1 and A83‐01 from the RCC organoid culture medium potentially reduced the overgrowth of normal kidney organoids. The depletion of nonessential culture components from the RCC organoid culture medium greatly reduced the substantial cost.

RCC organoids retained the histological architecture, biomarker expression profile, genetic alterations, and transcriptomic characteristics of their corresponding tumours and contained TME cells found in the corresponding tumours. Single‐cell RNA‐seq analysis revealed both intra‐ and intertumoural heterogeneity in RCC organoids, and the enrichment in features of specific cell clusters may predict different prognoses. Gene expression study revealed that high expression of genes associated with stemness‐related properties predicted high rates of RCC organoid derivation, suggesting that RCC organoids may originate from tumour cells with stem‐like properties.

To evaluate the use of RCC organoids as a tool to guide precision medicine, we performed drug screens on established RCC organoids. Consistent with our expectations, most RCC organoids were resistant to conventional chemotherapy drugs, such as cisplatin, 5‐FU and gemcitabine. When we analysed the organoid responses to targeted agents, we found that several compounds targeting the AKT signalling pathway or the MEK/ERK signalling pathway demonstrated therapeutic potential for treating RCC. We also observed that mTOR signalling pathway inhibitors displayed promising inhibitory effects on the growth of several RCC organoid lines. This is in line with the findings from several clinical trials that treatment with mTOR inhibitors everolimus or temsirolimus could prolong survival in metastatic RCC patients.^43^, ^44^, ^45^, ^46^ It is worth noting that although AZD8055, everolimus and temsirolimus share the same drug target, their effects on RCC organoids derived from different donors revealed striking differences, highlighting the necessity of choosing the appropriate treatment regimens. In addition, the inter‐ and intratumoural heterogeneity revealed by scRNA‐seq results may explain the differential responses of RCC organoids to these targeting agents. RCC organoids from different patients were composed of cell subclusters in which signalling pathways were differentially activated, and this may account for the differential responses to targeted drugs.

It is worth noting that tumour organoid models established in the present study did not preserve the TME such as stroma and immune cells, which limited their use in assessing the efficacy of antiangiogenic drugs and immune checkpoint inhibitors. Thus, there is an urgent need to develop organoid systems to integrate TME into these models.

RCC organoid‐based drug screenings not only facilitate personalized medicine but also promote the development of algorithms that accurately predict drug sensitivity. Correlating histology, WES, and RNA‐seq data with drug sensitivities by RCC organoids will hopefully help further our understanding of RCC carcinogenetic mechanisms. Previous reports demonstrate that tumour organoids derived from patients correlate with patients’ responses.^24^, ^47^, ^48^ Next, we will perform co‐clinical trials to test whether in vitro patient‐derived RCC organoids’ responses recapitulate patients’ responses to the corresponding treatments in vivo.

CD70 was highly expressed in RCC and, therefore, held great potential to act as a suitable target antigen for CAR‐T therapy. Using the RCC organoid model, the efficacy of CD70‐specific CAR‐T cells could be assessed. These RCC organoid models provide a platform to assess CAR‐T cells’ efficacy, which may advance the development of cancer immunotherapies for RCC.

In conclusion, RCC organoids preserve the characteristics of their original tumour tissues and may advance the basic research of RCC and promote the development of precision medicine.

## 4 MATERIALS AND METHODS

### 4.1 Ethical approval

All patients provided informed written consent for sample use. This study was approved by the Research Ethics Committee of Shenzhen Second Peoples’ Hospital and was conducted according to the guidelines of the local law.

### 4.2 Sample collection and tissue processing

Fresh RCC tissues, adjacent normal kidney tissues and matched peripheral blood were collected from patients who underwent radical nephrectomy or partial nephrectomy in Shenzhen Second People's Hospital. Clinical data of the RCC samples are summarized in Table S1.

Peripheral blood samples were aliquoted, snap‐frozen for DNA isolation and WES, and the results were used as a reference. RCC tumour samples and normal kidney samples were cut into small pieces for DNA and RNA isolation, histology analysis, cell isolation and organoid derivation.

### 4.3 Organoid culture

Each RCC tissue was split for organoid derivation, histology, DNA isolation and RNA isolation. RCC tumour and normal kidney tissues for organoid derivation were minced into small pieces and were subjected to enzymatic digestion in 5 ml of collagenase II (5 mg/ml) with ROCK inhibitor Y‐27632 dihydrochloride (10 μM) for 1 h at 37°C in a water bath shaker. Samples were centrifuged at 200 *g* for 5 min. After removing the supernatant, the digested tissues were incubated in 5 ml of TrypLE Express (in DPBS/1‐mM EDTA) for 5 min at 37°C. About 10 ml of AdDMEM/F12 supplemented with 20% FBS was added to the digestion suspension to neutralize trypsinization. After centrifugation, cells were suspended in AdDMEM/F12 supplemented with 20% FBS and were pipetted up and down to further dissociate tissue fragments. Cell suspensions were filtered through 70‐μm cell strainers prior to centrifugation. Cell pellets were resuspended in cold organoid culture medium and mixed with cold Matrigel (Matrigel should be >75% in the final solution), and 20 000 cells in 40‐μl droplets were deposited into prewarmed 6‐well plates. Cell culture plates were put upside down in the incubator for 10 min, and organoid culture medium was added. The composition of the RCC organoid culture medium can be found in Table S2. RCC organoids were passaged every 2–3 weeks with a split ratio of 1:2–1:3. Remove Y‐27632 from the culture medium from day 7 after initial plating.

The passage of organoids was conducted using a previously published protocol with minor modifications.^49^, ^50^, ^51^ Organoids were collected, centrifuged and incubated with TrypLE Express (in DPBS/1‐mM EDTA) with Y‐27632 (10 μM) for 5 min at 37°C. AdDMEM/F12 supplemented with 20% FBS was added before centrifugation at 200 g for 5 min. Organoids were resuspended in AdDMEM/F12 and pipetted up and down in AdDMEM/F12 about 10 times. After centrifugation, cells were resuspended in cold Matrigel for culture. The cryopreservation of organoids was conducted as previously described.^52^

### 4.4 HE staining and immunofluorescence staining

Tissues and organoids (>passage 5) were fixed in 10% neutral buffered formalin, dehydrated, embedded in paraffin and sectioned.

H&E staining and immunofluorescence staining were conducted on 4‐μm sections of tissue samples and organoids, using a published protocol with minor modifications.^53^ Paraffin sections were dewaxed, rehydrated and washed. For immunofluorescence staining, slides were incubated in boiled citrate buffer for 20 min for antigen retrieval. Slides were then blocked in 5% BSA in PBS and incubated with primary and secondary antibodies (listed in Table S3). Nuclei were stained with 4′,6‐diamidino‐2‐phenylindole. Immunofluorescence images were acquired using a confocal microscope.

### 4.5 Whole‐exome sequencing and genomic analysis

Genomic DNA was isolated from tissue samples, organoids (>passage 5) and blood with an AllPrep DNA/RNA Mini Kit (Qiagen). DNA libraries were created using Agilent SureSelect Human All Exon V6 kit (Agilent Technologies). The sequencing was performed on Illumina NovaSeq. Low‐quality reads and adaptors were removed using Fastp (v0.12.6).^54^ GATK (v4.1.9) was utilized to analyse single‐nucleotide variant (SNVs).^55^ Reads were mapped to the human reference genome (hg38) with the Burrows–Wheeler Alignment tool.^56^ Sequenced Reads number and mapping quality of WES data were included in Table S4. Mutect2 (default options) was implemented to analyse SNVs and indels in organoids and tumours. CNAs were analysed with TitanCNA (1.30.0).^57^ The effects of mutations were predicted using VEP (release 101).^58^

### 4.6 RNA sequencing and analysis

Total RNA was extracted organoids (>passage 5) and tissue samples with the AllPrep DNA/RNA Mini Kit (Qiagen). NEBNext Ultra RNA Library Prep Kit for Illumina (NEB, USA) was used for library preparation. RNA‐seq was performed on Illumina NovaSeq. Sequence reads were aligned to the Ensembl hg38 using STAR (v2.4.0j).^59^ Low‐quality and adaptor polluted reads were removed using fastp (0.20.0) by Novogene Company. Sequenced Reads number and mapping quality of RNA‐seq data were included in Table S5. RSEM was applied to analyse gene expression.^60^ GSEA and DAVID enrichment analyses were performed using clusterProfiler (v4.0.5).^61^ The pathway enrichment scores were calculated by GSVA method using GSVA (1.38.2) package in R^62^ with gene sets downloaded from the GSEA (v7.2) official website. Differential pathway enrichment analysis of Figure S6G was done using Limma (3.46.0) package in R.^63^ Differential expression analysis at gene levels was done using edgeR (3.34.1).^64^ Clinical information and expression matrix of RCC cohorts from TCGA database was downloaded using TCGAbiolinks (2.20.1).^65^ The uni/multivariate Cox regression analyses were conducted using ‘survival’ package in R using default parameters.

### 4.7 scRNA‐seq

RCC organoids (passage 4) were digested into single cells, and dead cells were eliminated using a Dead Cell Removal Kit (Miltenyi Biotec). All libraries were sequenced on the NovaSeq 6000 platform. BCL files were demultiplexed with the 10× Genomics i7 index using Illumina's bcl2fastq and mkfastq command from 10× Genomics CellRanger v4.0.0 tools. Extracted paired‐end FASTQ files were aligned with the genome (hg38), and the raw expression matrix was generated using the count function of CellRanger V4.0.

The raw unique molecular identifier (UMI) count matrix was analysed with Seurat3 script in R.^66^ Cells with nCount_RNA < 10 000, nFeature_RNA < 1000 or mitochondrial‐derived UMI counts >15% were considered low‐quality cells and were filtered out. Sequenced Reads number and mapping quality of scRNA‐seq data are included in Table S6. Doublet cells were identified using DoubletFinder script in R^67^ and were also filtered out. After this quality control, 29 100 genes in 18 584 cells were detected. Approximately 5000–8400 cells from each sample and a median of 3902 genes and 16 288 transcripts were captured per cell. The classic Seurat analysis pipeline was used for data analysis. In brief, UMI matrices were log_2_ normalized, and the 3500 most variable genes were used for analysis. PCA with the top 70 PCs was used for dimensionality reduction, followed by UMAP and tSNE. Cells were clustered using the *K*‐nearest neighbour graph‐based methods. Batch effect between patients was removed using harmony package integrated in SeuratWrappers.^68^ Cell types were identified using SingleR, and RCC data from Matthew D. Young, with known cell type messages, were used as a reference.^69^, ^70^ DEGs were analysed using FindMarkers script in Seurat package. CellCycleScoring in Seurat was used to perform cell cycle analysis. Pathway enrichment analysis was done by using VISION.^71^ Gene regulatory network of the ccRCC sample was done using pySCENIC using raw scRNA‐seq UMI data.^72^ Monocle 2 was employed to simulate the single‐cell trajectories using default parameters.^73^

### 4.8 Drug screening

The drug screening process was conducted on RCC organoids as previously described.^74^ In brief, RCC organoids (>passage 5) were released from Matrigel and filtered through 70‐μm cell strainers. Then, organoids were seeded in ultra‐low attachment 96‐well plates (Mingao biotechnology) in organoid medium containing 2% Matrigel. Organoid medium containing 6 concentrations (fivefold serial dilution) of each drug or DMSO controls, was added 1 day after plating. The maximal concentration of each drug was indicated in Table S7. The number of viable cells was measured using CellTiter‐Glo 3D (Promega) after 6 days. Dose‐response curves, IC_50_ values, and AUC values were calculated using GraphPad Prism 7. Drug testing was performed for three biological replicates (RCC organoids at different passages) with technical duplicates.

### 4.9 Generation of CD70 CAR‐T cells

The CAR was composed of full‐length human CD27 (CD70 receptor), the signalling domain of the costimulatory molecule 4‐1BB and CD3‐zeta chain. A lentiviral vector containing the CAR structure was cotransfected into 293FT cells with packing plasmids to generate lentiviral particles.

Peripheral blood mononuclear cells were obtained from healthy volunteers. CD3^+^ T cells were purified using CD3 magnetic beads, and purified T cells were incubated with CD3/CD28 beads before use. T cells were maintained in vivo 15 media (Lonza, Basel, Switzerland) containing 10% FBS, 50 IU/ml IL‐2 and 1 ng/ml IL‐15. T cells were infected with CAR lentivirus.

### 4.10 Co‐culture of CAR‐T cells with RCC organoids

RCC organoids (>passage 5) were cocultured with CAR‐T cells as previously reported.^36^ Briefly, RCC organoids were harvested, digested using TrypLE Express, seeded on a Matrigel layer in the organoid medium and cultured for 1 day. The medium was then changed to X‐VIVO 15 medium, and CAR‐T cells were added. After the incubation, coculture supernatant was harvested, and the release of TNF‐α and IFN‐γ was measured by ELISA (R&D). The apoptosis of organoids after coculture was determined using a Cleaved Caspase‐3 (Asp175) ELISA Kit (Abcam).

### 4.11 CFSE assay of T cell proliferation

Before coculture with RCC or normal kidney organoids, T cells were stained with CFSE (BioGems) for 20 min at 37°C. After coculture with organoids for 3 days, T cells were collected, purified using CD3 beads and subjected to flow cytometry.

### 4.12 Statistical analysis

Statistical analyses were performed using GraphPad Prism 7. Unless otherwise specified, all summary data are presented as mean ± standard error of the mean. Student's *t*‐test was adopted to compare the differences between two groups. Statistical significance was as follows: ns, not significant; *p < .05; **p < .01; ***p < .001.

## CONFLICT OF INTEREST

The authors declare that there is no conflict of interest that could be perceived as prejudicing the impartiality of the research reported.

## Supporting information

Supporting Information

## Data Availability

The raw sequence data reported in this paper were deposited in the Genome Sequence Archive in the BIG Data Center, Beijing Institute of Genomics (BIG), Chinese Academy of Sciences, under accession number HRA002381 (WES), HRA002424 (bulk RNA‐seq) and HRA002423 (scRNA‐seq), which are publicly accessible at http://bigd.big.ac.cn/gsa‐human.
