# Supplementary material for: Patient‐derived renal cell carcinoma organoids for personalized cancer therapy
Source: Clin Transl Med. 2022 Jul 8;12(7):e970. doi: 10.1002/ctm2.970 (PMC9270001; doi:10.1002/ctm2.970)
Supplement: Supplementary file 1 — Supporting Information [file CTM2-12-e970-s001.docx]

**SUPPORTING INFORMATION**

**Supplemental figures**


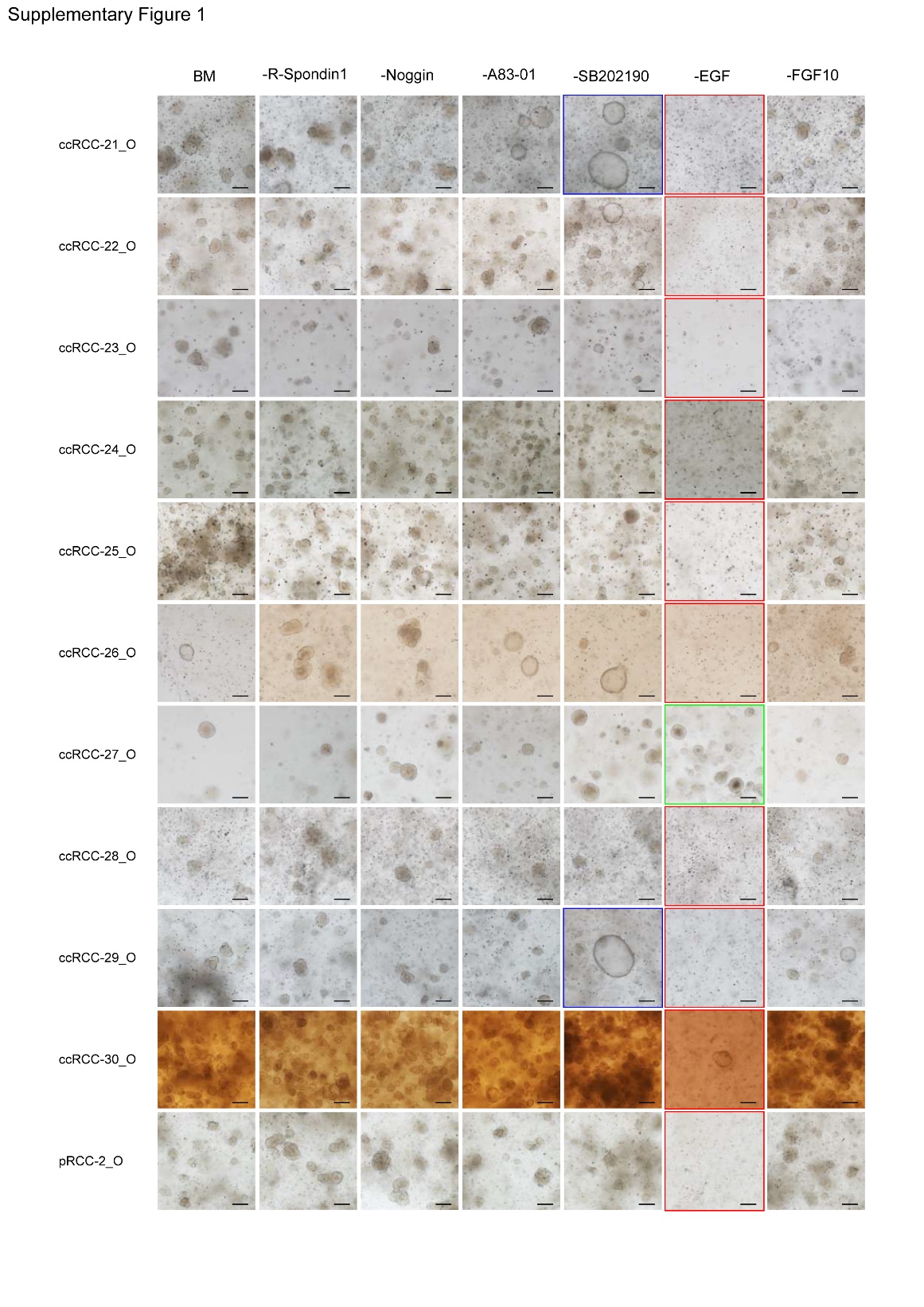


**Figure S1. Optimization of the medium for the culture of RCC organoids**. Shown are bright-field images of RCC organoids formed in basal medium (BM) modified medium (with each component individually omitted from the BM) after 2 weeks of culture in indicated media, Scale bar, 100 μm.


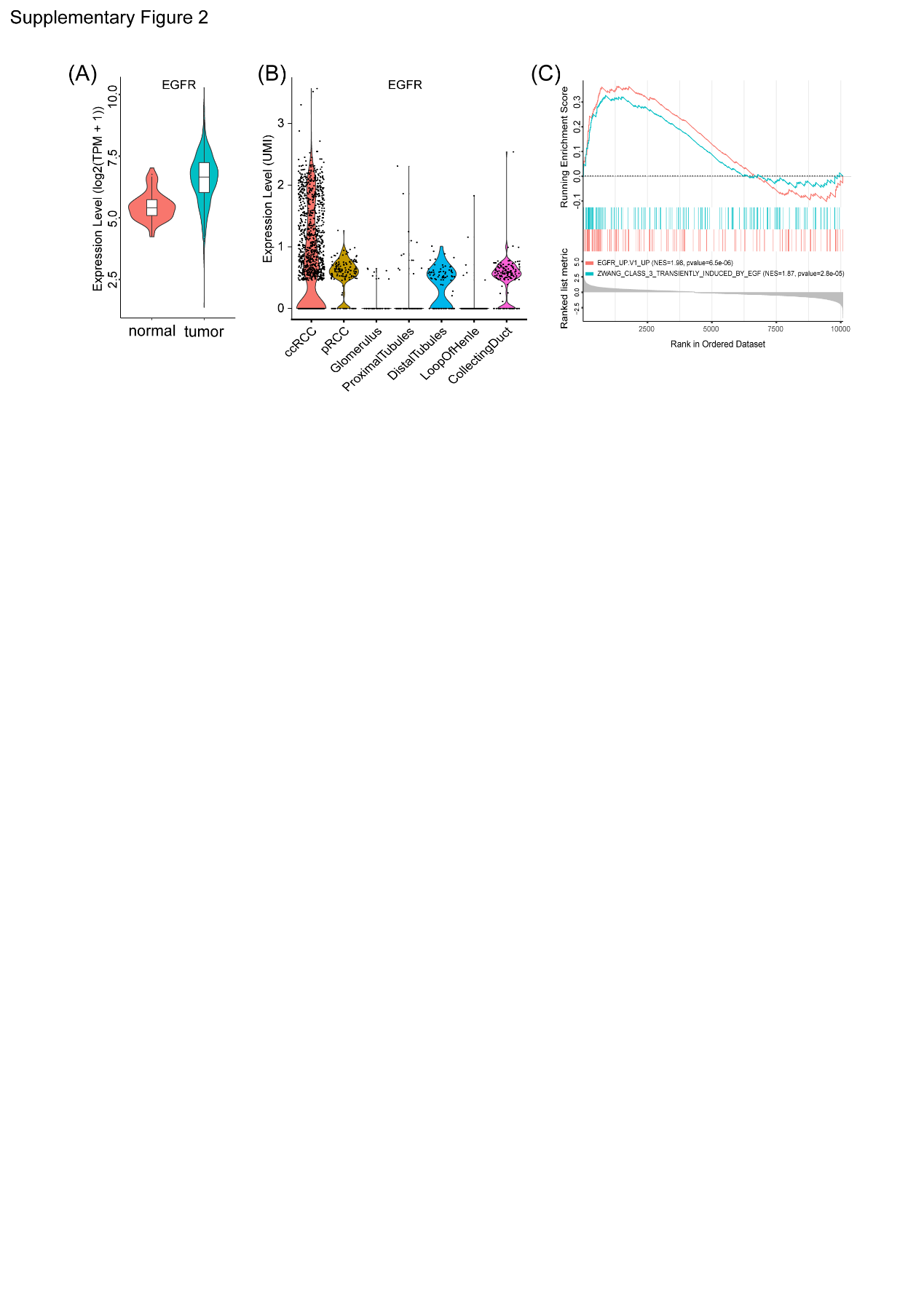


**Figure S2. The expression pattern of EGFR and the enrichment of EGFR pathways in RCC. (**A) The expression levels of EGFR in normal kidney tissues and RCC tissues using TCGA KIRC data. (B) The expression of EGFR at single cell level using the adult RCC patients from Matthew D. Young.^1^ (C) The enriched EGFR associated pathways calculated by clusterProfiler between normal ductal cells and carcinoma cells using single cell RNA-seq data from Matthew D. Young.


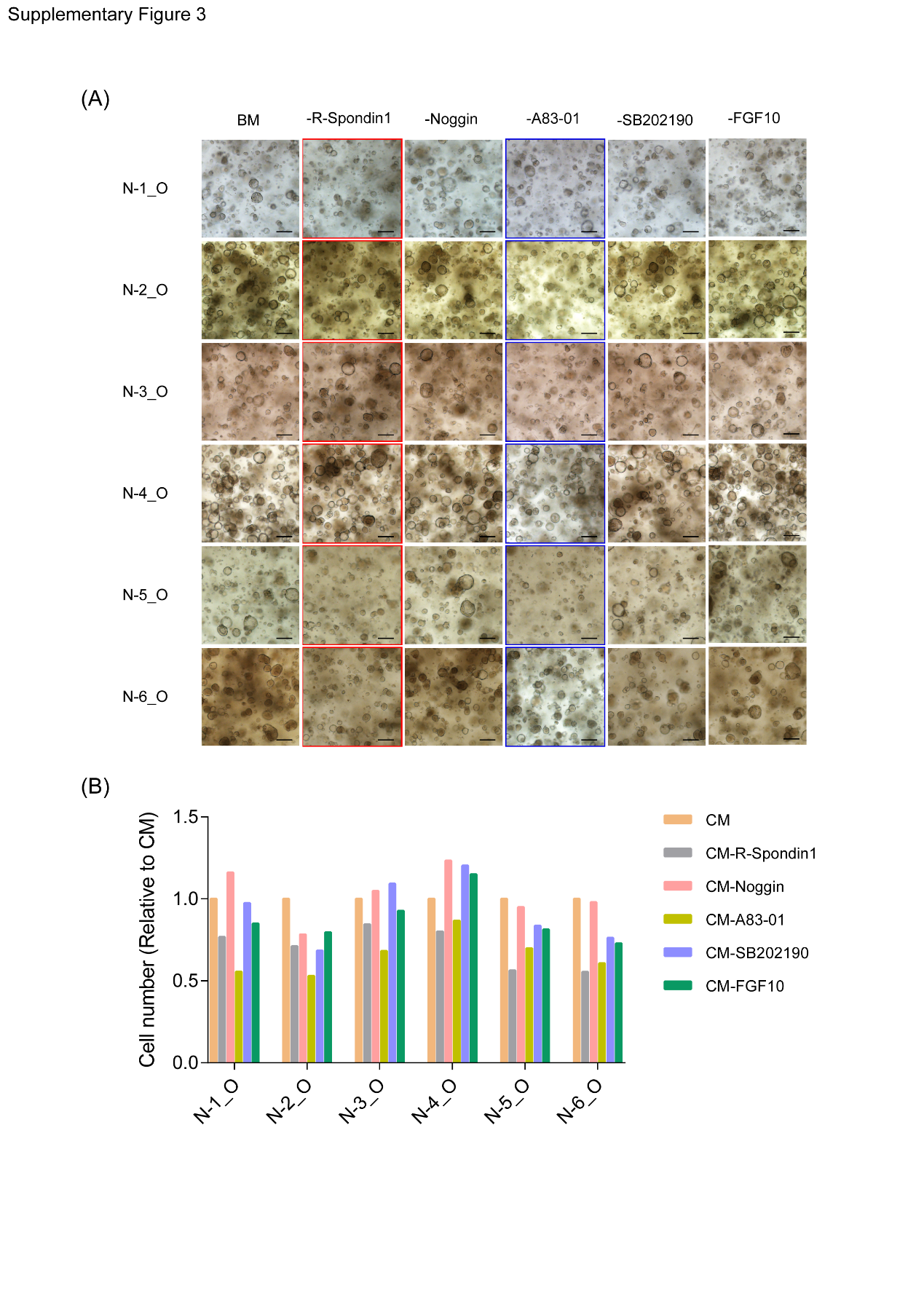


**Figure S3. The niche factor requirements by normal kidney organoids.** Bright-field images (A) and relative cell number (B) of normal kidney organoids formed in basal medium (BM) modified medium (with each component individually omitted from the BM) after 2 weeks of culture in indicated media, Scale bar, 100 μm.


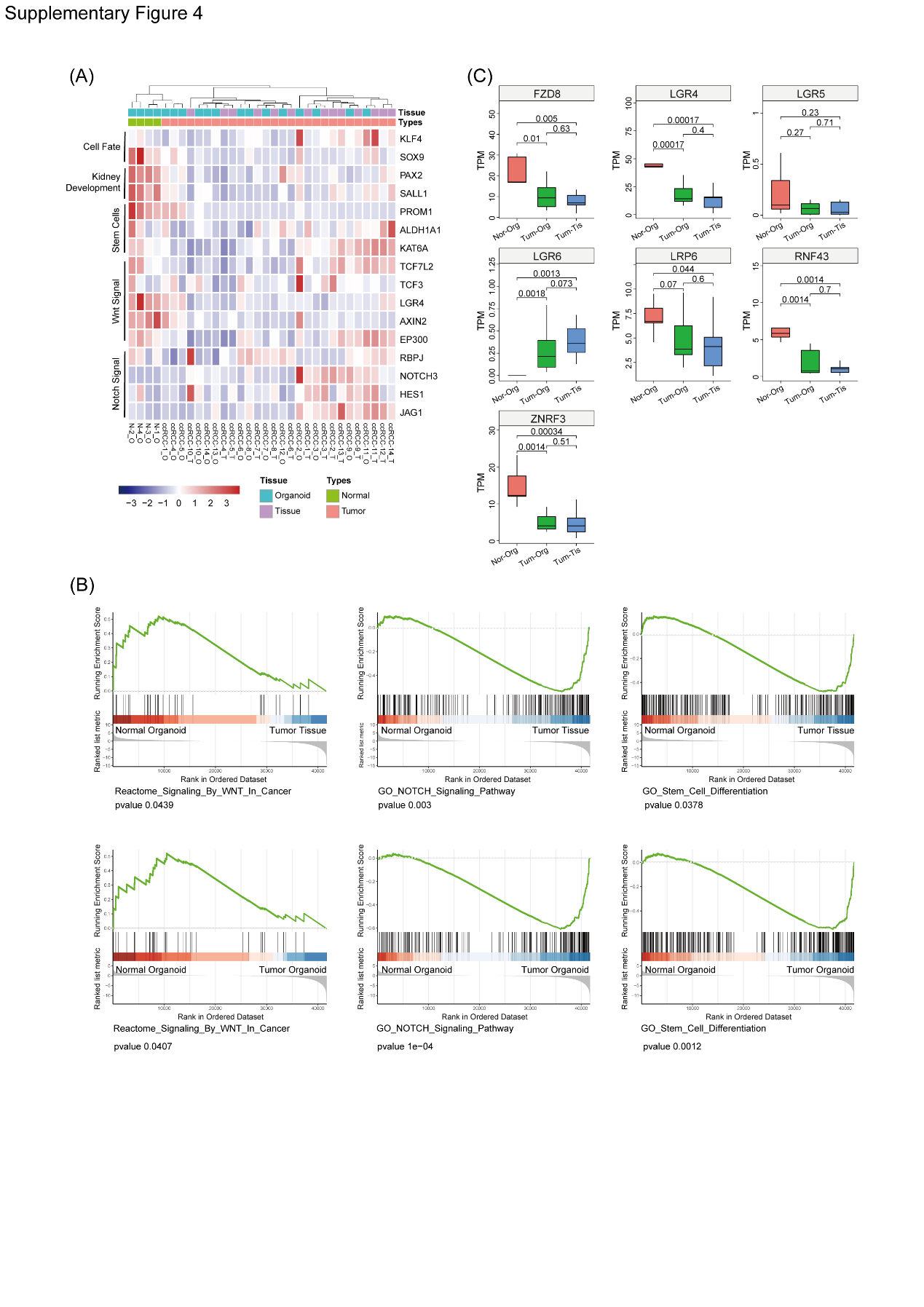


**Figure S4. Higher activation of WNT and stemness signaling in normal kidney organoids. (**A) Heatmap showed the expression pattern of genes associated with stemness or kidney development signaling pathways reported in a previous study.^2^ Expression values were row scaled. (B) GSEA analysis showed higher activation of WNT signaling pathways and lower activation of differentiation signaling pathways in normal kidney organoids. (C) Boxplot showed the expression pattern of RSPO1 receptors. Nor-Org, Tum-Org and Tum-Tis represented for normal organoids, tumor organoids and tumor tissues. Wilcox test was used for differential expression analysis.


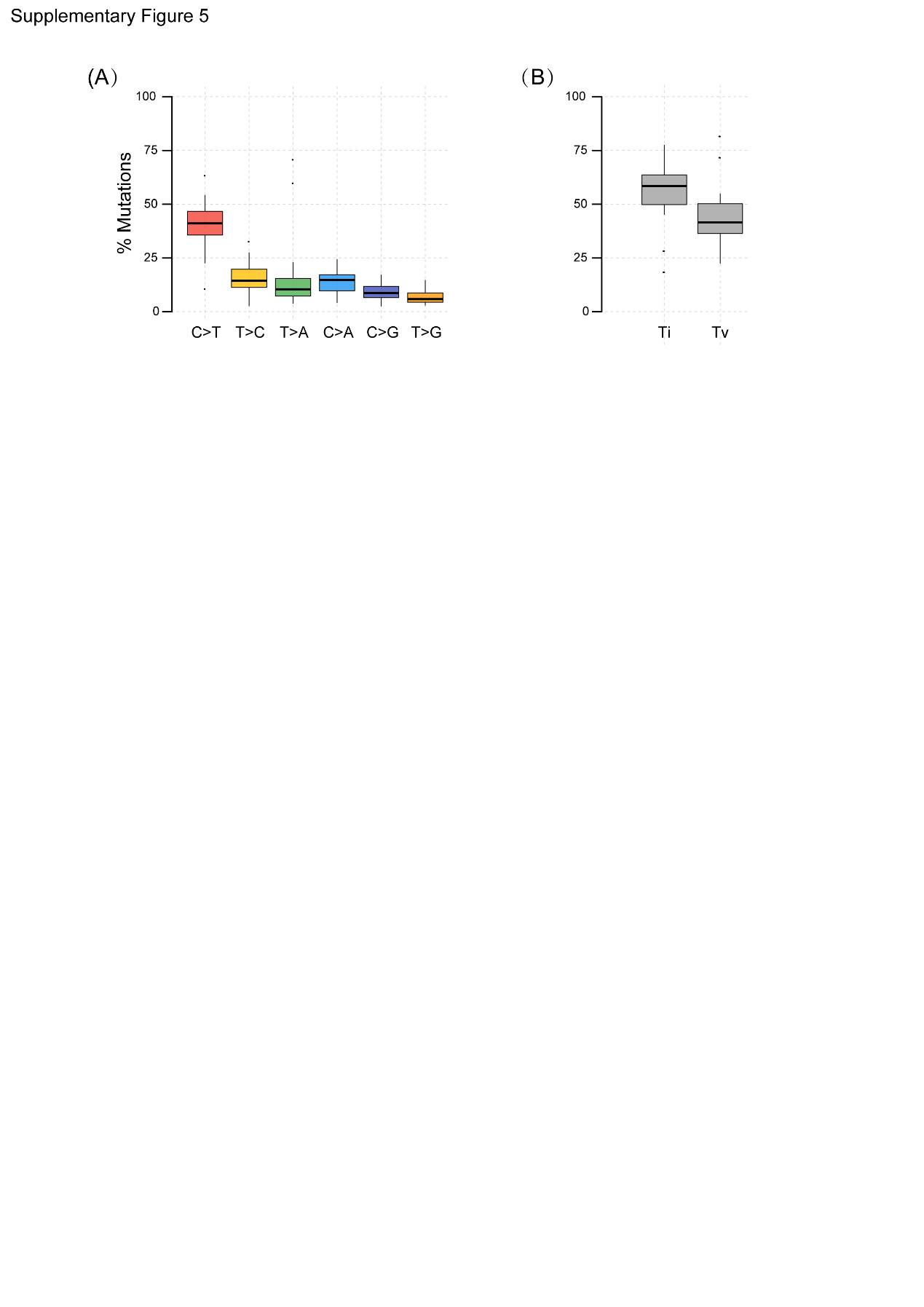


**Figure S5. Base substitutions in RCC organoids (_O) and parental tumor tissues (_T).** Percentage of the six types of base substitutions (A) and the transitions and transversions spectrum (B) across all samples. Graphs shown are mean±SD.


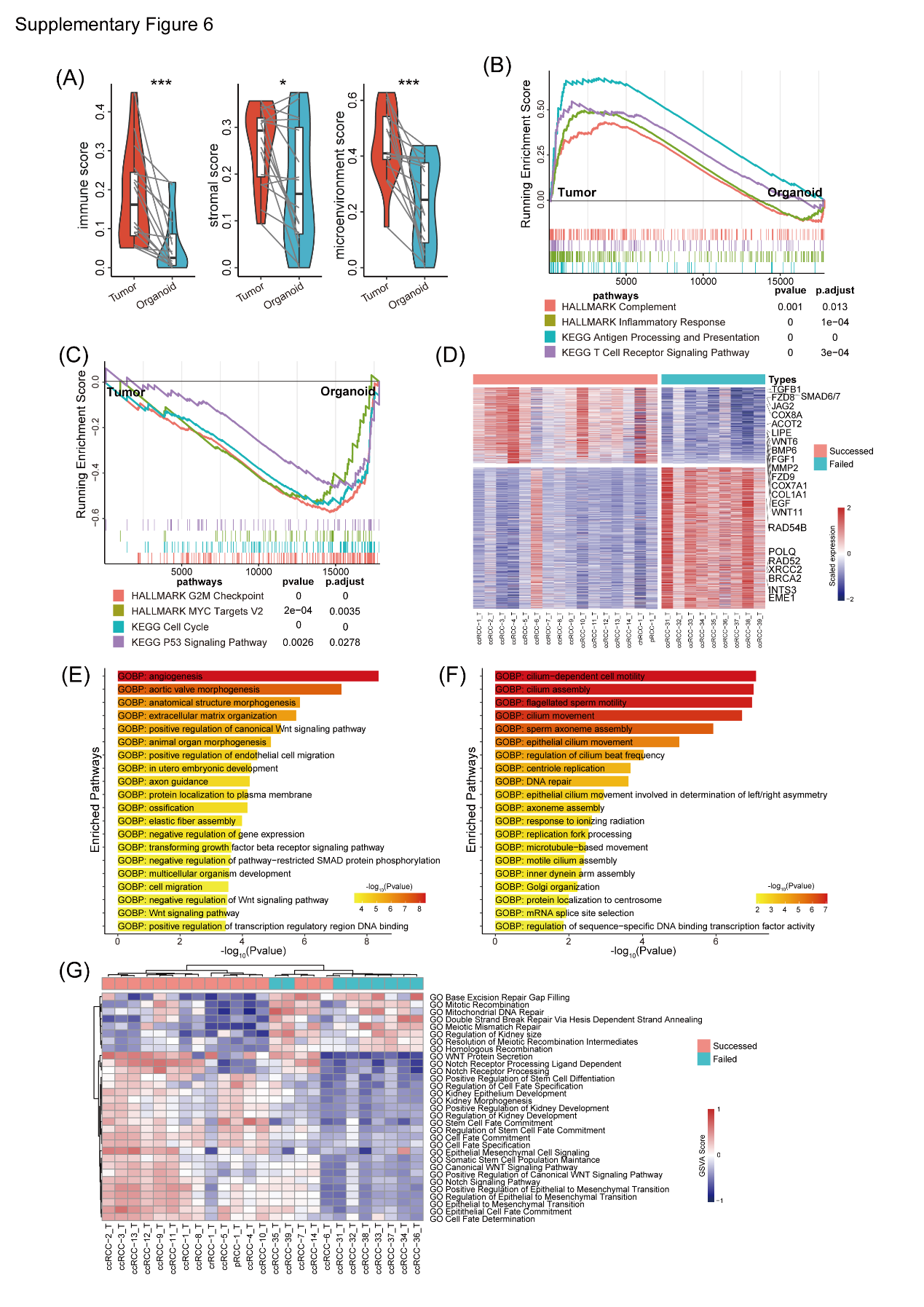


**Figure S6. Transcriptomic analysis of RCC tissues and organoids. (**A) Boxplot showed the difference between RCC tissues and organoids in immune score, stromal score, and microenvironment score. Wilcoxon test was used for differential test. (B, C) GSEA plot showed the enrichment of immune-associated pathways (B) and cell cycle-associated pathways (C) between RCC tissues and organoids. (D) Heatmap showed the differentially expressed genes between 16 RCC samples which successfully formed organoids and 10 RCC samples that failed to derive tumor organoids. Genes with |log_2_FC| >1 and adjust *p*-value <0.05 were presented. 1078 genes and 2007 genes were presented in thr upper and lower panel, respectively. (E, F) Boxplot showed the top 20 significantly enriched pathways in 16 RCC samples which successfully formed organoids (E) and 10 RCC samples that failed to derive tumor organoids (F). (G) Heatmap showed the part of the most differentially enriched pathways between RCC samples which successfully formed organoids and RCC tissues which failed to derive tumor organoids calculated by GSVA with *p*-value＜0.01.


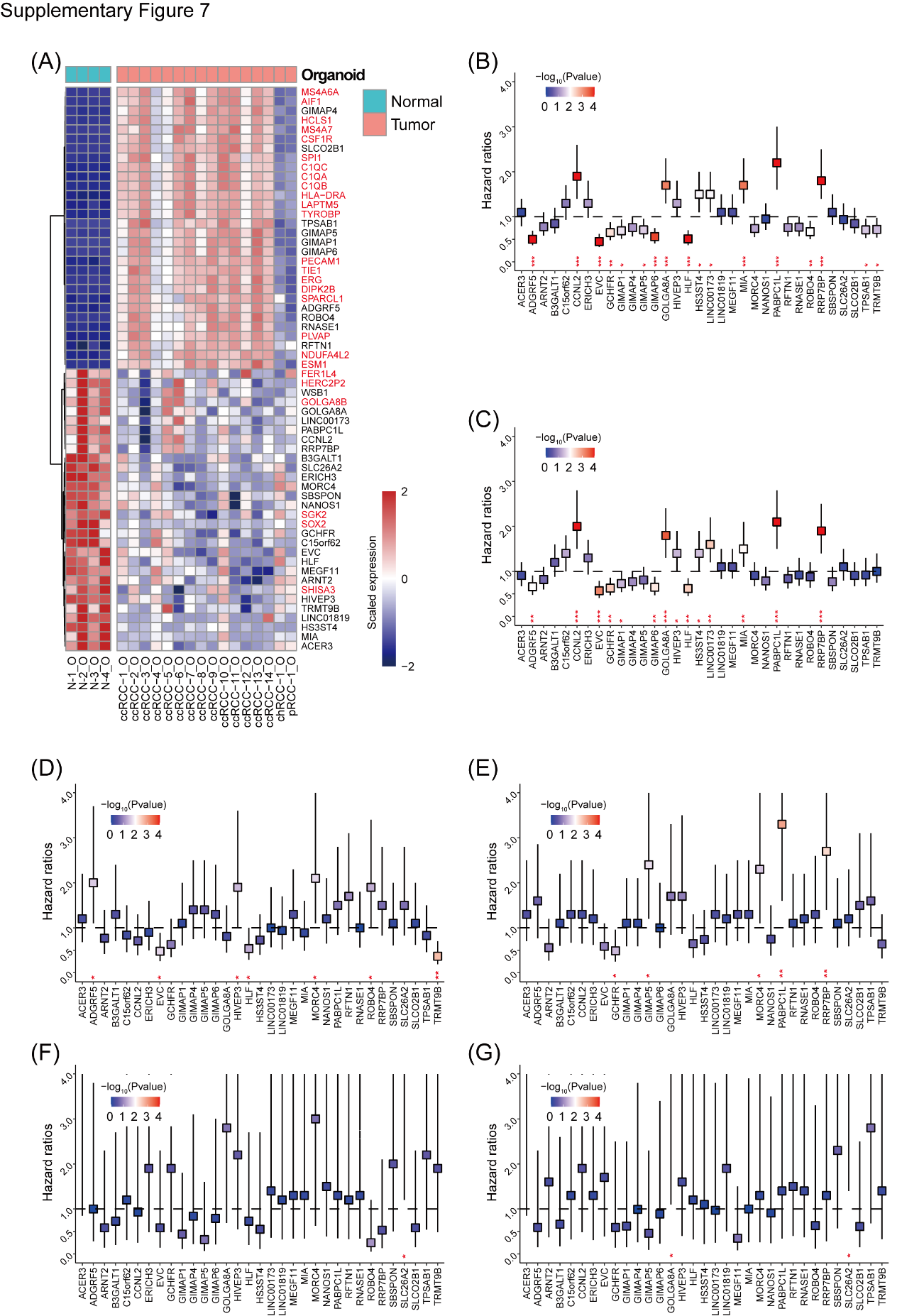


**Figure S7. RCC organoids as a platform to identify prognostic tumor biomarkers.** (A) heatmap of 60 differentially expressed genes between RCC organoids and normal kidney organoids identified by RNA-seq data analysis. Each column represents one sample, and each row represents one gene. Red indicates high expression; green indicates low expression. Genes marked in red were reported to be differentially expressed by others. (B-G) Overall survival analysis based on cox proportional-hazards model using publicly available TCGA data. Tumor samples were grouped into expression-hi and -low groups by median TPM value. Uni-cox regression analysis of KIRC, KIRP and KCH cohort were presented in (B), (D), and (F), respectively. Multi-cox regression analysis included genes expression and tumor stage of KIRC, KIRP and KCH cohort were presented in (C), (E), and (G), respectively. Genes were significantly associated with prognosis were labeled with red star. Y-axis showed the HR score (HR >1 meant the gene were associated with poor prognosis when highly expressed, HR <1 meant the gene were associated with good prognosis when highly expressed).


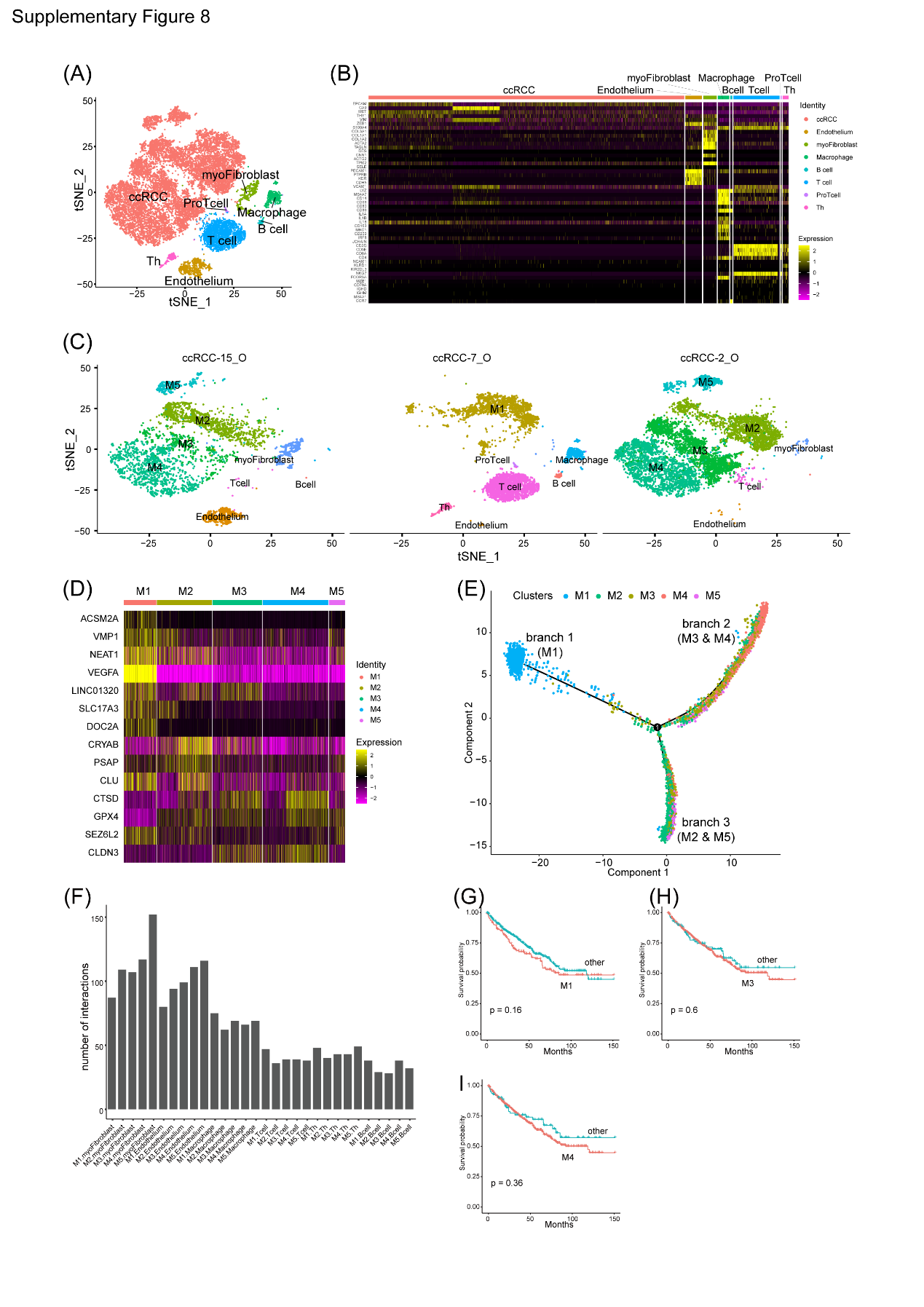


**Figure S8. The cellular heterogeneity in RCC organoids. (**A) tSNE plot of 18584 cells from 3 RCC organoid lines. (B) Heatmap showed the expression profiles of well-known marker genes for each subcluster. (C) tSNE plot showed the distribution of each subcluster in RCC organoid lines. (D) Heatmap showed the marker genes specific for TP1 and TP2 clusters which reported by Kevin, et al.^3^ (E) Trajectory analysis of RCC cells with each color coded for clusters using Monocle2. (F) The number of cell-type-specific ligand-receptor interactions inferred by CellPhoneDB between each carcinoma subcluster and other microenvironment cells. Kaplan-Meier analysis of overall survival (OS) in TCGA cohorts separated by M1 (G), M3 (H), M4 (I) signature using SingleR script.


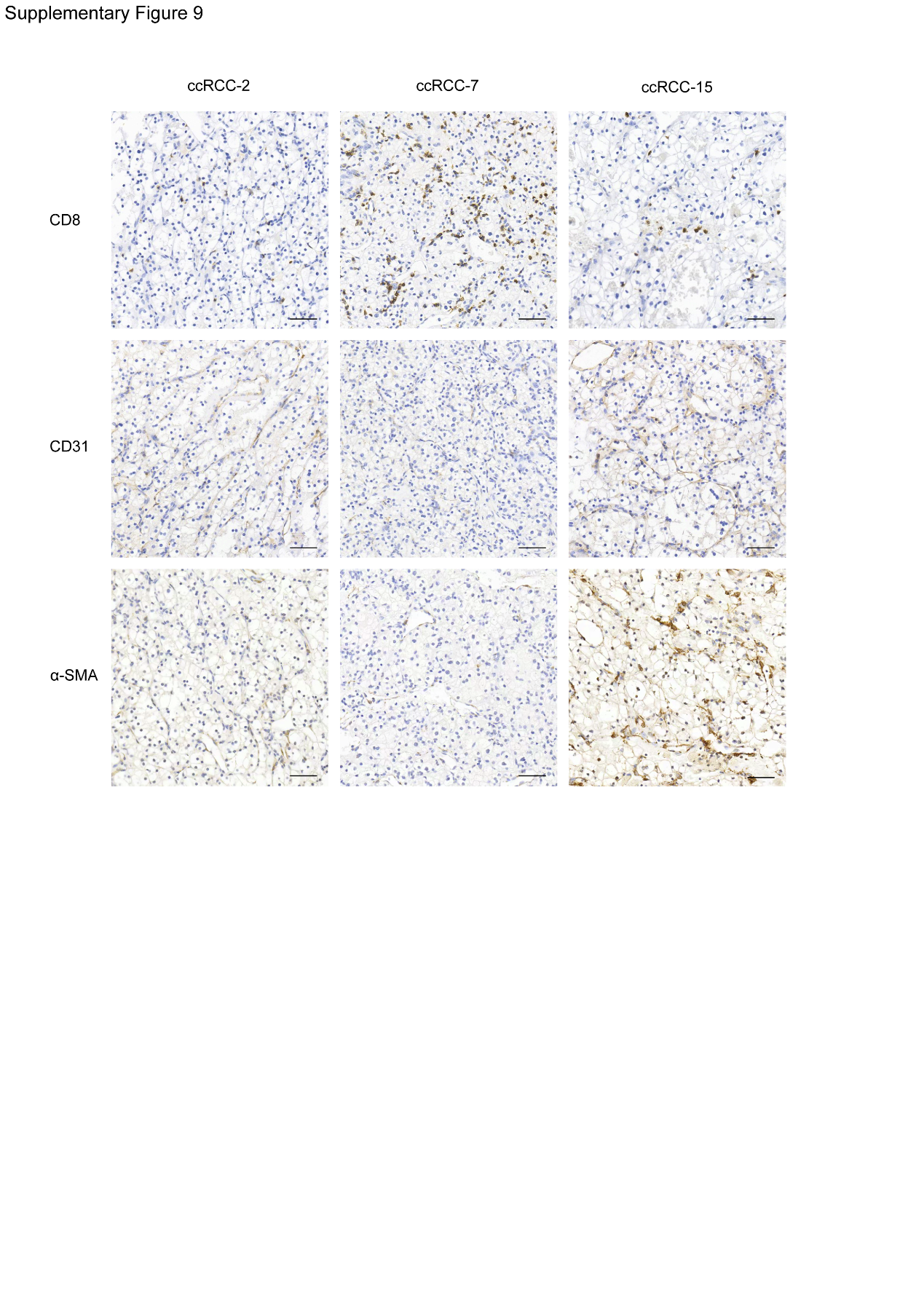


**Figure S9. Immunohistochemistry analysis of the expression of marker genes in 3 RCC tissues.** Shown is the expression of CD8, CD31, and α-SMA in tumor tissues of ccRCC-2, ccRCC-7, and ccRCC-15. Scale bar, 50 μm.


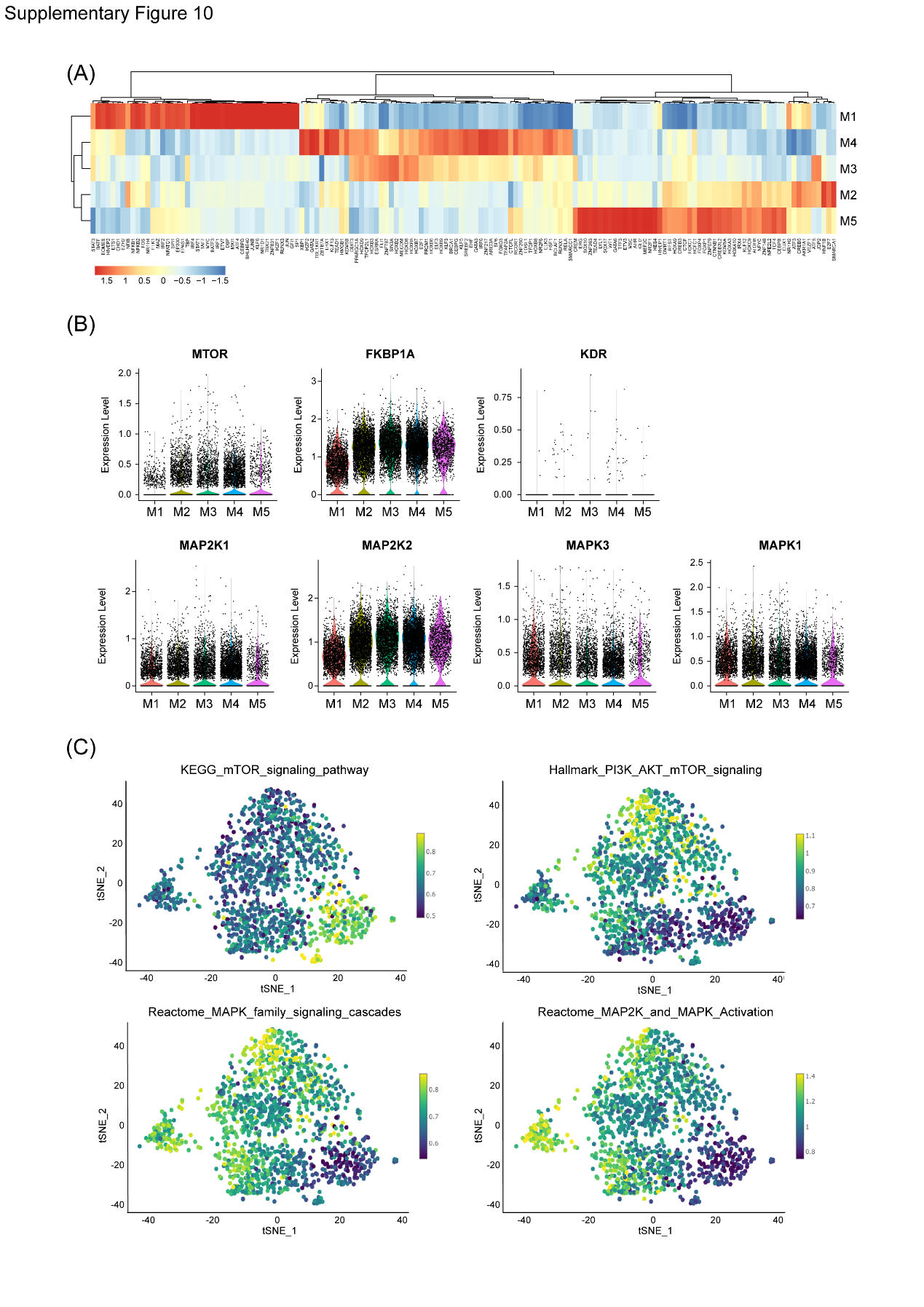


**Figure S10. The expression pattern of candidate therapeutic target genes at single cell level.** (A) The activities of regulons in each cluster calculated by SCENIC. Only those regulators with average activity larger than 1 in any cluster were showed. (B) The expression pattern of several therapeutic target genes associated with mTOR, RTKi, MEK or ERK signaling pathway. (C) The enrichment scores of mTOR and MAPK pathways in RCC cells calculated by VISON.


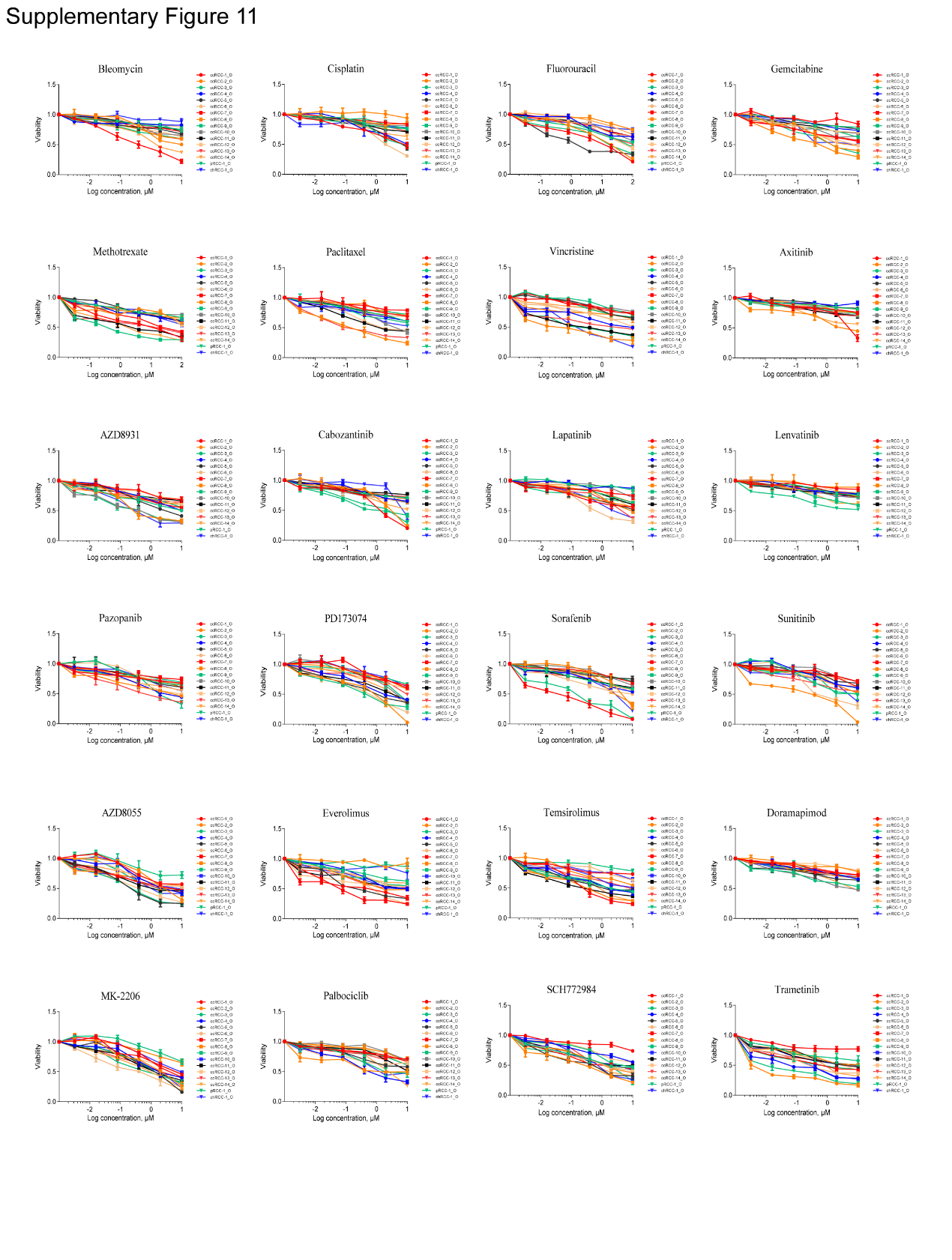


**Figure S11. Drug response analyses of RCC organoids, related to Figure 6.** Shown are the dose response curves of 24 compounds against 18 RCC organoid lines. Each data point represents three biological replicates, with error bars representing ± SEM.


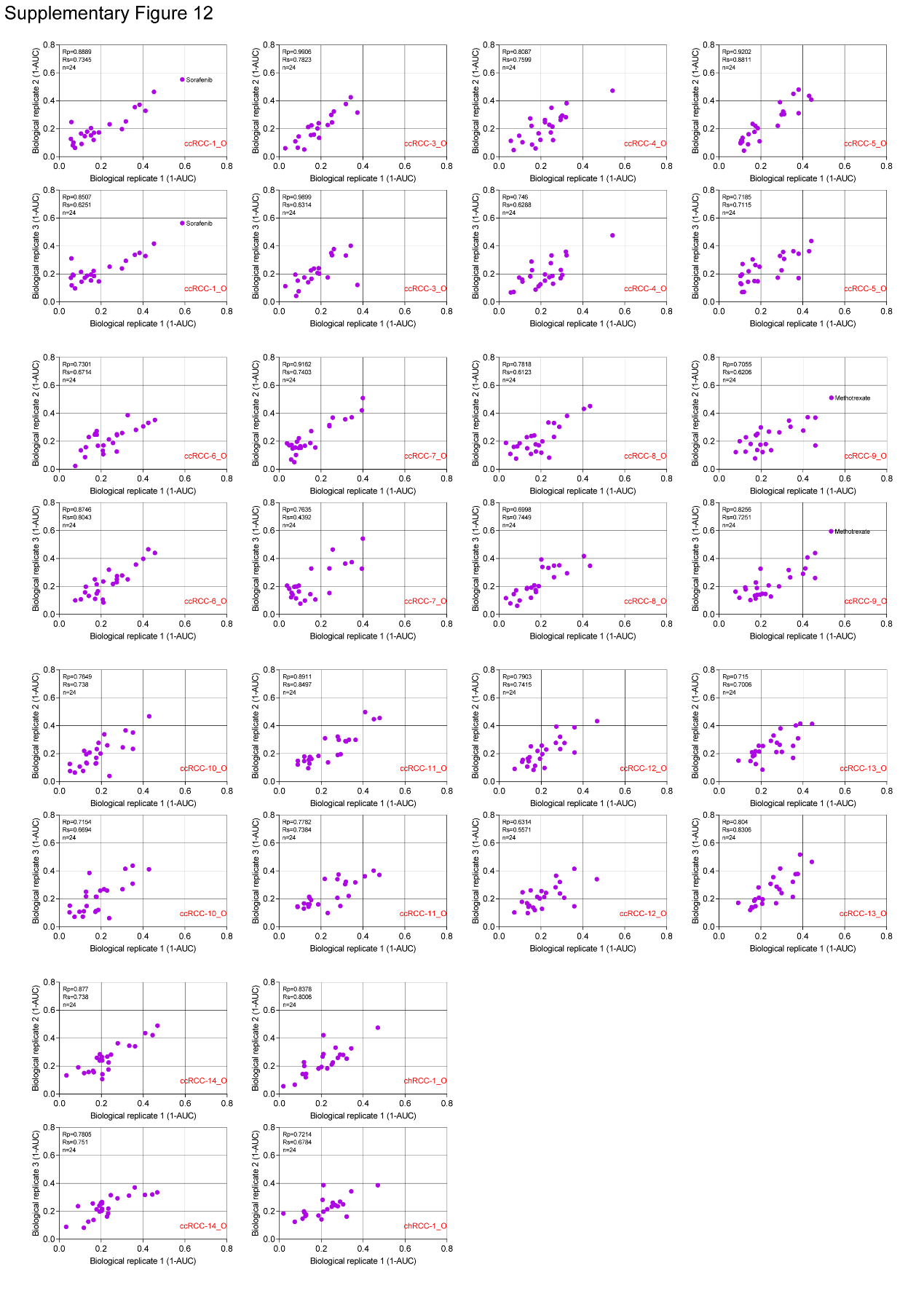


**Figure S12. The correlation of 1-AUC values from biological replicates, related to Figure 6.** Shown are the scatterplots of 1-AUC (area under the curve) values from two biological replicates of the drug screening data, highlighting drugs (red) having an obvious inhibitory effect on viability (1-AUC > 0.5 for both biological replicates) of indicated organoid lines.
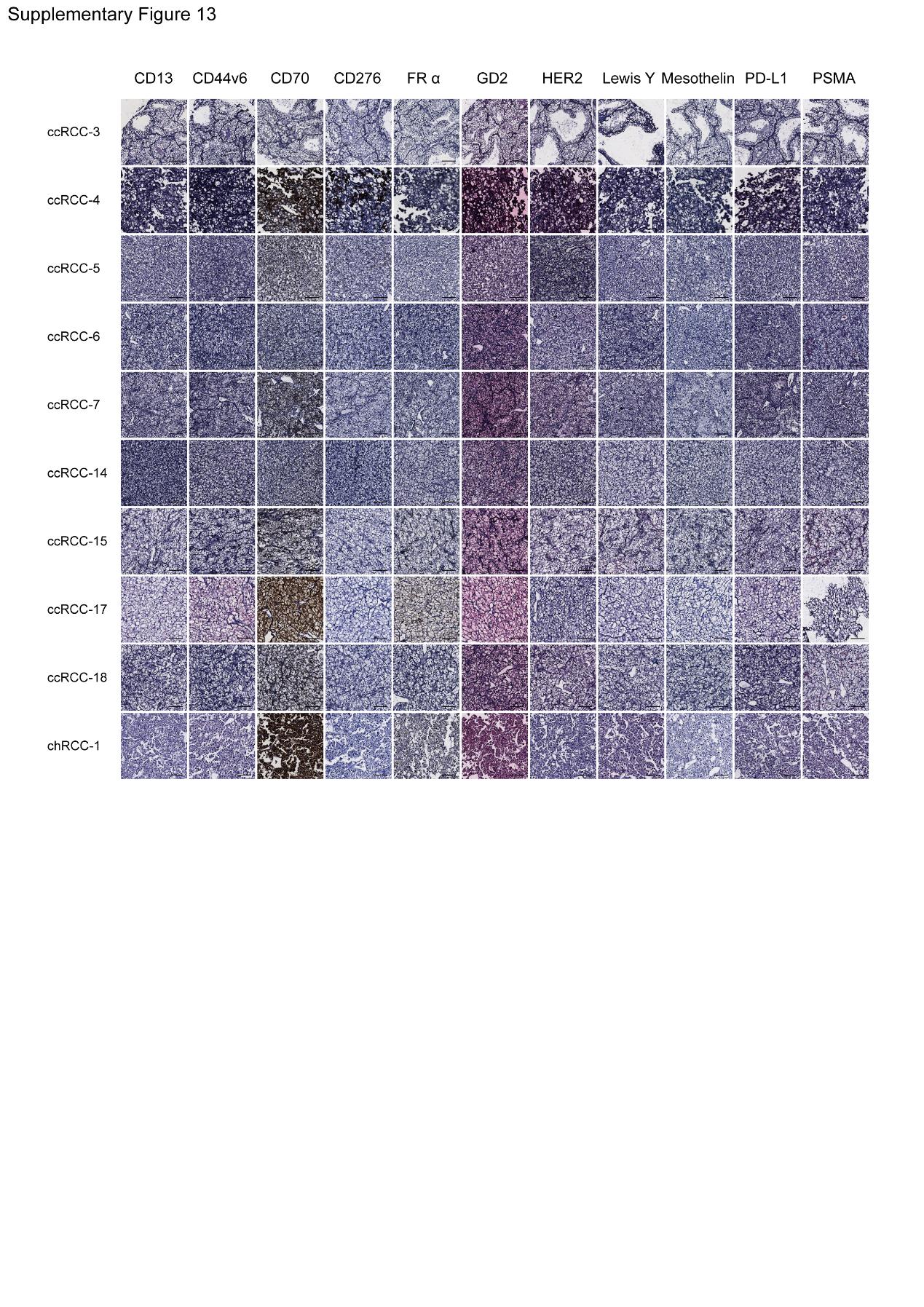


**Figure S13. Immunohistochemistry analysis of indicated surface antigens in RCC tissues, related to Figure 7.** Antibodies against identified CAR-recognizable targets in solid tumors, including CD13, CD44v6, CD70, CD276, FRα, GD2, HER2, Lewis Y, Mesothelin, PD-L1 and PSMA, were used as indicated. Scar bar, 100 μm.

**References**

1. Young MD, Mitchell TJ, Vieira Braga FA, et al. Single-cell transcriptomes from human kidneys reveal the cellular identity of renal tumors. *Science (New York, NY)*. Aug 10 2018;361(6402):594-599. doi:10.1126/science.aat1699

2. Fendler A, Bauer D, Busch J, et al. Inhibiting WNT and NOTCH in renal cancer stem cells and the implications for human patients. *Nature communications*. Feb 17 2020;11(1):929. doi:10.1038/s41467-020-14700-7

3. Bi K, He MX, Bakouny Z, et al. Tumor and immune reprogramming during immunotherapy in advanced renal cell carcinoma. *Cancer cell*. Mar 10 2021;doi:10.1016/j.ccell.2021.02.015

**Supplemental tables**

**Table S1. Patients' information.**

| Line | Age at  diagnosis | Gender | Race | Subtype | Location | Stage | Node  Status | Normal organoid |
| --- | --- | --- | --- | --- | --- | --- | --- | --- |
| ccRCC-1_O | 40 | M | Asian | ccRCC | Right kidney | II | N0 |  |
| ccRCC-2_O | 64 | M | Asian | ccRCC | Left kidney | II | N0 |  |
| ccRCC-3_O | 26 | M | Asian | ccRCC | Left kidney | II | N0 |  |
| ccRCC-4_O | 55 | M | Asian | ccRCC | Left kidney | III | N0 |  |
| ccRCC-5_O | 36 | F | Asian | ccRCC | Left kidney | I | N0 |  |
| ccRCC-6_O | 63 | F | Asian | ccRCC | Right kidney | II | N0 | N-3_O |
| ccRCC-7_O | 55 | F | Asian | ccRCC | Right kidney | II | N0 |  |
| ccRCC-8_O | 63 | M | Asian | ccRCC | Left kidney | III | N0 | N-4_O |
| ccRCC-9_O | 63 | M | Asian | ccRCC | Right kidney | II | N1 |  |
| ccRCC-10_O | 54 | F | Asian | ccRCC | Right kidney | I | N0 | N-5_O |
| ccRCC-11_O | 38 | M | Asian | ccRCC | Right kidney | I | NX |  |
| ccRCC-12_O | 69 | M | Asian | ccRCC | Right kidney | I | NX |  |
| ccRCC-13_O | 54 | M | Asian | ccRCC | Right kidney | I | NX |  |
| ccRCC-14_O | 45 | F | Asian | ccRCC | Left kidney | I | N0 |  |
| ccRCC-15_O | 68 | F | Asian | ccRCC | Left kidney | I | NX |  |
| ccRCC-16_O | 49 | M | Asian | ccRCC | Right kidney | I | N0 | N-6_O |
| ccRCC-17_O | 62 | F | Asian | ccRCC | Right kidney | IV | N0 | N-7_O |
| ccRCC-18_O | 57 | M | Asian | ccRCC | Right kidney | II | N0 |  |
| ccRCC-19_O | 53 | M | Asian | ccRCC | Left kidney | I | N0 |  |
| ccRCC-20_O | 68 | M | Asian | ccRCC | Left kidney | II | N0 |  |
| ccRCC-21_O | 46 | M | Asian | ccRCC | Right kidney | I | N0 | N-8_O |
| ccRCC-22_O | 46 | M | Asian | ccRCC | Right kidney | II | N0 | N-9_O |
| ccRCC-23_O | 73 | F | Asian | ccRCC | Right kidney | II | N0 |  |
| ccRCC-24_O | 64 | M | Asian | ccRCC | Left kidney | I | N0 |  |
| ccRCC-25_O | 71 | M | Asian | ccRCC | Right kidney | II | N0 |  |
| ccRCC-26_O | 64 | F | Asian | ccRCC | Right kidney | II | N0 |  |
| ccRCC-27_O | 63 | M | Asian | ccRCC | Left kidney | III | N0 |  |
| ccRCC-28_O | 53 | M | Asian | ccRCC | Right kidney | II | N0 |  |
| ccRCC-29_O | 63 | M | Asian | ccRCC | Left kidney | II | N0 |  |
| ccRCC-30_O | 68 | M | Asian | ccRCC | Left kidney | III | N0 |  |
| pRCC-1_O | 31 | F | Asian | pRCC | Right kidney | II | N0 |  |
| pRCC-2_O | 26 | M | Asian | pRCC | Right kidney | III | N0 |  |
| chRCC-1_O | 68 | M | Asian | chRCC | Right kidney | I | N0 | N-10_O |
|  |  |  |  |  |  |  |  |  |
| Failed cases in organoid derivation |  |  |  |  |  |  |  |  |
| ccRCC-31 | 48 | M | Asian | ccRCC | Right kidney | I | N0 |  |
| ccRCC-32 | 68 | M | Asian | ccRCC | Left kidney | II | N0 |  |
| ccRCC-33 | 56 | F | Asian | ccRCC | Right kidney | II | N0 | N-1_O |
| ccRCC-34 | 38 | M | Asian | ccRCC | Right kidney | II | N0 |  |
| ccRCC-35 | 47 | M | Asian | ccRCC | Right kidney | I | N0 |  |
| ccRCC-36 | 61 | M | Asian | ccRCC | Right kidney | II | N0 | N-2_O |
| ccRCC-37 | 53 | M | Asian | ccRCC | Left kidney | II | N0 |  |
| ccRCC-38 | 63 | F | Asian | ccRCC | Left kidney | III | N0 |  |
| ccRCC-39 | 44 | F | Asian | ccRCC | Right kidney | I | N0 |  |
| pRCC-3 | 61 | M | Asian | pRCC | Left kidney | II | N0 |  |

**Table S2. Organoid media recipe.**

| RCC organoid media recipe | | | |
| --- | --- | --- | --- |
| Reagent Name | Supplier | Catalogue  number | Final Concentration in Media |
| Advanced DMEM/F-12 | Thermo Fisher Scientific | 12634-010 | 1x |
| Antibiotic-Antimycotic | Thermo Fisher Scientific | 15240-062 | 1x |
| GlutaMAX™ Supplement | Thermo Fisher Scientific | 35050-061 | 1x |
| HEPES | Thermo Fisher Scientific | 15630-080 | 10 mM |
| B-27™ Supplement (50X),  serum free | Gibco | 17504-044 | 1x |
| N-Acetylcysteine | Sigma | A9165 | 1.25 mM |
| Nicotinamide | Sigma | N0636 | 10 mM |
| SB202190 | Sigma | S7076 | 10 mM |
| Y-27632 | Abmole Bioscience | M1817 | 10 mM |
| Human EGF | Peprotech | AF-100-15 | 50 ng/mL |
|  |  |  |  |
| Normal kidney organoid  media recipe | | | |
| Reagent Name | Supplier | Catalogue  number | Final Concentration in Media |
| Advanced DMEM/F-12 | Thermo Fisher Scientific | 12634-010 | 1x |
| Antibiotic-Antimycotic | Thermo Fisher Scientific | 15240-062 | 1x |
| GlutaMAX™ Supplement | Thermo Fisher Scientific | 35050-061 | 1x |
| HEPES | Thermo Fisher Scientific | 15630-080 | 10 mM |
| B-27™ Supplement (50X),  serum free | Gibco | 17504-044 | 1x |
| N-Acetylcysteine | Sigma | A9165 | 1.25 mM |
| Nicotinamide | Sigma | N0636 | 10 mM |
| SB202190 | Sigma | S7076 | 10 mM |
| A83-01 | Sigma | SML0788 | 500 nM |
| Recombinant Human  R-spondin 1 Protein | bioGenous technologies | RWL003 | 500 μg/L |
| Human Noggin | Peprotech | 120-10C-1000 | 100 μg/L |
| Y-27632 | Abmole Bioscience | M1817 | 10 mM |
| Recombinant Human  FGF-10 | Peprotech | 100-26 | 20 μg/L |
| Human EGF | Peprotech | AF-100-15 | 50 ng/mL |

**Table S3. Antibody information.**

| Primary antibodies | | | | |
| --- | --- | --- | --- | --- |
| Antigen | Supplier | Catalogue number | Origin | Dilution |
| CK7 | Thermo Fisher | 180234 | mouse | 1/100 |
| AMACR | Sigma-Aldrich | HPA020912 | rabbit | 1/200 |
| CD10 | Maixin Biotech | MAB-0668 | rabbit | 1/100 |
| PAX2 | Maixin Biotech | RAB-0648 | mouse | 1/100 |
| E-Cadherin | Cell Signaling Technology | 3195 | rabbit | 1/100 |
| CK8/18 | Maixin Biotech | MAB-0650 | mouse | 1/200 |
| Vimentin | Cell Signaling Technology | 5741 | rabbit | 1/100 |
| Ki67 | Maixin Biotech | MAB-0672 | mouse | 1/100 |
| α-SMA | Abcam | ab5694 | rabbit | 1/200 |
| CD31 | Abcam | ab28364 | rabbit | 1/100 |
| CD8 | Maixin Biotech | MAB-0021 | mouse | 1/100 |
|  |  |  |  |  |
| Secondary antibodies | | | | |
| Reagent Name | Supplier | Catalogue number | Origin | Dilution |
| Donkey anti-Rabbit IgG (H+L) Highly Cross-Adsorbed Secondary Antibody, Alexa Fluor 488 | Thermo Fisher Scientific | A21206 | donkey | 1/1000 |
| Goat anti-Mouse IgG (H+L) Cross-Adsorbed Secondary Antibody, Alexa Fluor 555 | Thermo Fisher Scientific | A21422 | goat | 1/1000 |

**Table S4. Sequenced reads number and mapping quality of WES data.**

| Samples | Total Paired Reads | Mapped Paired Reads | Average Sequenced Depths |
| --- | --- | --- | --- |
| ccRCC-1_B | 44,805,634 | 43,908,065 | 101.09 |
| ccRCC-1_O | 79,850,862 | 78,600,586 | 163.88 |
| ccRCC-1_T | 88,303,034 | 86,875,245 | 154.16 |
| ccRCC-2_B | 59,060,436 | 58,269,720 | 119.07 |
| ccRCC-2_O | 76,428,948 | 75,535,206 | 168.52 |
| ccRCC-2_T | 89,326,796 | 88,389,309 | 189.17 |
| ccRCC-3_B | 49,217,814 | 48,819,825 | 115.87 |
| ccRCC-3_O | 109,002,220 | 107,922,431 | 243.02 |
| ccRCC-3_T | 81,645,767 | 80,804,774 | 206.56 |
| ccRCC-4_O | 91,083,906 | 89,212,690 | 153.16 |
| ccRCC-4_T | 99,158,898 | 98,159,747 | 242.11 |
| ccRCC-5_B | 52,915,678 | 52,495,982 | 116.50 |
| ccRCC-5_O | 103,714,889 | 102,545,160 | 217.27 |
| ccRCC-5_T | 83,608,916 | 82,774,971 | 196.82 |
| ccRCC-6_B | 42,657,945 | 42,172,474 | 95.69 |
| ccRCC-6_O | 84,135,317 | 83,456,217 | 216.05 |
| ccRCC-6_T | 85,893,093 | 85,238,564 | 222.74 |
| ccRCC-7_B | 68,393,148 | 67,869,445 | 159.01 |
| ccRCC-7_O | 86,824,151 | 86,329,538 | 216.90 |
| ccRCC-7_T | 78,488,704 | 77,816,459 | 195.29 |
| ccRCC-8_B | 37,646,316 | 37,403,794 | 95.60 |
| ccRCC-8_O | 83,468,386 | 82,510,855 | 187.66 |
| ccRCC-8_T | 56,815,091 | 56,355,573 | 150.05 |
| ccRCC-9_O | 84,832,341 | 83,205,340 | 230.62 |
| ccRCC-9_T | 84,548,888 | 83,989,652 | 195.44 |
| ccRCC-10_B | 45,692,531 | 45,292,254 | 107.13 |
| ccRCC-10_O | 89,806,606 | 88,766,346 | 225.98 |
| ccRCC-10_T | 89,220,649 | 88,261,626 | 197.11 |
| ccRCC-11_B | 30,258,745 | 30,015,043 | 74.64 |
| ccRCC-11_O | 51,811,988 | 51,409,765 | 126.36 |
| ccRCC-11_T | 82,101,189 | 81,597,948 | 192.40 |
| ccRCC-12_B | 37,029,227 | 36,779,170 | 90.76 |
| ccRCC-12_O | 71,906,064 | 71,332,069 | 184.17 |
| ccRCC-12_T | 73,810,016 | 73,129,009 | 181.60 |
| ccRCC-13_B | 26,462,357 | 26,255,570 | 68.51 |
| ccRCC-13_O | 84,404,683 | 81,723,478 | 193.37 |
| ccRCC-13_T | 82,446,894 | 81,800,967 | 201.43 |
| ccRCC-14_B | 35,827,478 | 35,595,839 | 89.40 |
| ccRCC-14_O | 83,845,481 | 83,065,996 | 197.13 |
| ccRCC-14_T | 80,921,657 | 80,444,517 | 210.10 |
| chRCC-1_B | 46,971,674 | 46,019,234 | 106.92 |
| chRCC-1_O | 91,510,264 | 90,013,733 | 202.01 |
| chRCC-1_T | 87,332,730 | 85,836,750 | 186.65 |
| pRCC-1_B | 42,187,467 | 41,837,775 | 97.48 |
| pRCC-1_O | 91,936,845 | 90,922,678 | 226.24 |
| pRCC-1_T | 94,044,215 | 93,332,582 | 222.46 |

_T, tumor tissue; _O, organoids; _B, blood;

**Table S5. Sequenced reads number and mapping quality of RNA-seq data.**

| Samples | Total Paired Reads | Uniquely Mapped Paired Reads | Exon region mapped Paired Reads |
| --- | --- | --- | --- |
| ccRCC-1_O | 41,497,884 | 37,708,270 | 34,155,349 |
| ccRCC-1_T | 45,721,246 | 41,178,996 | 37,074,807 |
| ccRCC-2_O | 58,283,345 | 52,025,825 | 46,867,061 |
| ccRCC-2_T | 56,900,473 | 50,445,981 | 44,995,554 |
| ccRCC-3_O | 48,462,144 | 44,203,189 | 39,746,931 |
| ccRCC-3_T | 53,563,503 | 47,442,179 | 42,551,948 |
| ccRCC-4_O | 44,438,334 | 40,959,837 | 36,732,041 |
| ccRCC-4_T | 46,954,097 | 42,730,200 | 38,731,960 |
| ccRCC-5_O | 43,179,575 | 36,121,385 | 31,513,415 |
| ccRCC-5_T | 44,157,801 | 37,710,305 | 33,805,503 |
| ccRCC-6_O | 46,812,278 | 43,006,619 | 37,854,776 |
| ccRCC-6_T | 49,525,097 | 45,602,700 | 37,948,492 |
| ccRCC-7_O | 45,996,870 | 41,842,691 | 37,139,135 |
| ccRCC-7_T | 46,533,120 | 42,313,049 | 37,508,008 |
| ccRCC-8_O | 39,007,997 | 35,981,331 | 32,071,738 |
| ccRCC-8_T | 43,976,487 | 38,374,397 | 34,038,877 |
| ccRCC-9_O | 45,038,322 | 40,598,019 | 36,167,000 |
| ccRCC-9_T | 40,656,627 | 37,466,648 | 33,459,939 |
| ccRCC-10_O | 51,080,744 | 46,139,640 | 41,471,779 |
| ccRCC-10_T | 53,468,461 | 45,302,525 | 40,259,715 |
| ccRCC-11_O | 38,417,676 | 35,279,181 | 31,069,096 |
| ccRCC-11_T | 35,003,907 | 32,233,296 | 28,693,481 |
| ccRCC-12_O | 43,802,240 | 40,472,268 | 35,357,200 |
| ccRCC-12_T | 34,769,043 | 32,173,730 | 28,810,722 |
| ccRCC-13_O | 43,781,059 | 39,486,847 | 35,430,321 |
| ccRCC-13_T | 40,736,628 | 37,574,955 | 33,758,194 |
| ccRCC-14_O | 41,464,717 | 37,767,179 | 33,676,795 |
| ccRCC-14_T | 47,915,131 | 43,891,393 | 38,841,839 |
| ccRCC-31_T | 39,232,154 | 35,859,901 | 30,892,470 |
| ccRCC-32_T | 40,245,044 | 37,299,596 | 32,039,412 |
| ccRCC-33_T | 42,000,454 | 39,184,710 | 32,945,930 |
| ccRCC-34_T | 29,650,192 | 27,180,197 | 23,021,461 |
| ccRCC-35_T | 40,015,253 | 37,483,823 | 31,872,210 |
| ccRCC-36_T | 31,286,444 | 28,516,318 | 23,944,245 |
| ccRCC-37_T | 48,344,425 | 45,587,609 | 38,934,163 |
| ccRCC-38_T | 29,298,887 | 26,801,632 | 21,154,713 |
| ccRCC-39_T | 56,926,515 | 53,477,046 | 45,445,591 |
| chRCC-1_O | 45,780,105 | 41,482,633 | 37,697,903 |
| chRCC-1_T | 48,914,212 | 44,757,194 | 40,688,529 |
| pRCC-1_O | 55,878,782 | 51,458,525 | 46,124,503 |
| pRCC-1_T | 43,040,742 | 35,215,568 | 31,609,455 |
| N-1_O | 37,801,586 | 34,736,287 | 31,192,347 |
| N-2_O | 44,160,630 | 40,739,061 | 34,412,119 |
| N-3_O | 46,890,599 | 43,106,401 | 38,573,461 |
| N-4_O | 44,528,088 | 41,019,474 | 36,616,218 |

_T, tumor tissue; _O, organoids;

**Table S6. Sequenced reads number and mapping quality of scRNA-seq data.**

| Samples | Total number of sequenced reads per sample | Totally Estimated Number of Cells | Mean Reads per Cell before QC | Median Genes per Cell before QC | Median Genes per Cell after QC | Median Transcripts per Cell after QC |
| --- | --- | --- | --- | --- | --- | --- |
| ccRCC-2_O | 433,137,536 | 11,442 | 37,855 | 3,487 | 3814 | 16045 |
| ccRCC-7_O | 420,403,861 | 8,028 | 52,367 | 1,852 | 2450 | 7255 |
| ccRCC-15_O | 439,716,337 | 9,320 | 47,180 | 3,798 | 4962.5 | 21249.5 |

_O, organoids;

**Table S7. List of drugs screened.**

| Drug Name | Supplier | Catalogue number | Target | Max  Concentration (μM) |
| --- | --- | --- | --- | --- |
| Bleomycin | Selleck Chemicals | S1214 | DNA replication | 10 μM |
| Cisplatin | Sigma | 1134357 | DNA replication | 10 μM |
| Fluorouracil | Selleck Chemicals | S1209 | DNA replication | 100 μM |
| Gemcitabine | Sigma | 1288463 | DNA replication | 10 μM |
| Methotrexate | Sigma | M9929 | DHFR | 100 μM |
| Paclitaxel | Selleck Chemicals | S1150 | Tubulin | 10 μM |
| Vincristine | Selleck Chemicals | S1241 | Tubulin | 10 μM |
| Axitinib | Selleck Chemicals | S1005 | VEGFR,c-KIT,  PDGFR | 10 μM |
| AZD8931 | Selleck Chemicals | S2192 | EGFR, ErbB2, ErbB3 | 10 μM |
| Cabozantinib | Selleck Chemicals | S4001 | c-Met/VEGFR2 | 10 μM |
| Lapatinib | Selleck Chemicals | S2111 | EGFR/HER22 | 10 μM |
| Lenvatinib | Selleck Chemicals | S1164 | VEGFR1, VEGFR2,  VEGFR3 | 10 μM |
| Pazopanib | Selleck Chemicals | S1035 | c-KIT, FGFR, PDGFR | 10 μM |
| PD173074 | Selleck Chemicals | S1264 | FGFR1 | 10 μM |
| Sorafenib | Selleck Chemicals | S7397 | VEGFR/PDGFR | 10 μM |
| Sunitinib | Selleck Chemicals | S1042 | VEGFR2/PDGFR | 10 μM |
| AZD8055 | Selleck Chemicals | S1555 | mTOR | 10 μM |
| Everolimus | Sigma | E-068 CERILLIANT | mTOR | 10 μM |
| Temsirolimus | Selleck Chemicals | S1044 | mTOR | 10 μM |
| Doramapimod | Selleck Chemicals | S1574 | p38 MAPK | 10 μM |
| MK-2206 | Selleck Chemicals | S1078 | AKT1/2/3 | 10 μM |
| Palbociclib | Selleck Chemicals | S1579 | CDK4/CDK6 | 10 μM |
| SCH772984 | Selleck Chemicals | S7101 | ERK1/2 | 10 μM |
| Trametinib | Selleck Chemicals | S2673 | MEK1/2 | 10 μM |
